# Synthesis and Characterization of Cobalt(III), Nickel(II) and Copper(II) Mononuclear Complexes with the Ligand 1,3-bis[(2-aminoethyl)amino]-2-propanol and Their Catalase-Like Activity

**DOI:** 10.1371/journal.pone.0137926

**Published:** 2015-09-17

**Authors:** Bianca M. Pires, Daniel M. Silva, Lorenzo C. Visentin, Bernardo L. Rodrigues, Nakédia M. F. Carvalho, Roberto B. Faria

**Affiliations:** 1 Instituto de Química, Universidade Federal do Rio de Janeiro, Rio de Janeiro, Rio de Janeiro, Brazil; 2 NanoBusiness Informação e Inovação Ltda., Rio de Janeiro, Rio de Janeiro, Brazil; 3 Departamento de Química, Universidade Federal de Minas Gerais, Belo Horizonte, Minas Gerais, Brazil; University of Edinburgh, UNITED KINGDOM

## Abstract

In this work, we present the synthesis and characterization of two new mononuclear complexes with the ligand 1,3-bis[(2-aminoethyl)amino]-2-propanol (HL), [Co(L)(H_2_O)](ClO_4_)_2_ (1), [Ni(HL)](ClO_4_)_2_ (2), as well as the known complex [Cu(HL)](ClO_4_)_2_ (3) for comparison. Their abilities to catalyze the dismutation of H_2_O_2_ and the oxidation of cyclohexane were investigated. The complexes were characterized by X-ray diffraction, elemental analysis, electronic and infrared spectroscopy, cyclic voltammetry, electrospray ionization mass spectrometry (ESI-MS) and conductivity measurements. The X-ray structures showed that the nickel (2) and copper (3) complexes are tetracoordinated, with the metal ion bound to the nitrogen atoms of the ligand. On the other hand, the cobalt complex (1) is hexacoordinated, possessing additional bonds to the alkoxo group of the ligand and to a water molecule. Neither of the complexes was able to catalyze the oxidation of cyclohexane, but all of them exhibited catalase-like activity, following Michaelis-Menten kinetics, which suggest resemblance with the catalase natural enzymes. The catalytic activity followed the order: [Ni(HL)](ClO_4_)_2_ (2) > [Cu(HL)](ClO_4_)_2_ (3) > [Co(L)(H_2_O)](ClO_4_)_2_ (1). As far as we know, this is the first description of a nickel complex presenting a significant catalase-like activity.

## Introduction

The coordination between a metal ion and peroxide plays an important role in many biological systems [1]. Metalloenzymes such as methane monooxygenase (MMO) and catalase are two examples of dinuclear proteins known or believed to share a peroxide adduct during their catalytic cycle [2]. MMO is responsible for oxidizing methane into methanol at mild conditions, as well as others hydrocarbons and halocarbons [3]. The oxidation starts when a dioxygen molecule is activated in a two-electron oxidation process. On the other hand, catalase is responsible for the biological defense against hydrogen peroxide by its conversion to water and dioxygen (Eq 1). The dismutation process involves a two-electron transfer from the hydrogen peroxide to a diiron-peroxide adduct [4].

$$2\;H_{2}O_{2}\;\to 2\;H_{2}O\;+\;O_{2}$$ (1)

Two forms of active sites are found in MMO, a binuclear iron center in the soluble form of the enzyme [2] and mononuclear/binuclear copper centers in the protein-bound form [3]. Similarly, there are mostly two kinds of catalase enzymes, the most abundant possess an heme-type structures that contain an iron(III)-protoporphyrin IX prosthetic group in the active site, and can be found in almost all aerobic organisms. The second is a recently discovered class of manganese catalase that possesses a binuclear manganese center and occurs only in bacteria [5]. Lately, functional models have shown potential biomedical application as therapeutic agents against oxidative stress. Despite the natural enzymes possess iron or manganese in their active sites, complexes of other metals have being investigated as catalase models, such as copper [6–16], cobalt [15,17] and ruthenium [18].

In this work, we present the syntheses and characterization, including the X-ray crystal structure, of two new complexes with the ligand 1,3-bis[(2-aminoethyl)amino]-2-propanol (HL), [Co(L)(H_2_O)](ClO_4_)_2_ (**1**), [Ni(HL)](ClO_4_)_2_ (**2**). The known complex [Cu(HL)](ClO_4_)_2_ (**3**) [19, 20] was also studied for comparison. The catalase-like and MMO towards the oxidation of cyclohexane with H_2_O_2_, were also investigated. As far as we know, this is the first study revealing a significant catalase-like activity of a nickel complex.

## Experimental

### Materials and measurements

The solvents and reagents were used as received without further treatment. The ligand 1,3-bis[(2-aminoethyl)amino]-2-propanol (HL) was synthesized from epichlorohydrin and ethylenediamine, as previously described [21].

Infrared spectra were collected on a FTIR Nicolet Magna-IR 760 spectrophotometer (KBr or CsI pellets or film between NaCl window).

UV-Vis spectra were recorded on a Varian Cary 1E spectrophotometer in water solution. Infrared spectra were collected on a FTIR Nicolet Magna-IR 760 spectrophotometer (KBr or CsI pellets or film between NaCl window). Conductivity measurements were carried out with solutions containing 1.0×10^−3^ mol dm^-3^ of the complexes, using a BioCristal NT CVM conductivimeter, employing a conductivity cell CA150.

Electrospray ionization mass spectrometry (ESI-MS) measurements were performed on a high resolution ESI-TOF (micrOTOF, Bruker Daltonics, Bremen, Germany) mass spectrometer. The compound to be tested was dissolved in methanol prior to analysis. Full scans were acquired under the following conditions: capillary, 5.5 kV, capillary exit, 100 V. The spectra were obtained in positive-ion mode.

Cyclic voltammetry experiments were carried out in water using a BAS Epsilon potentiostat/galvanostat and a three-electrode system, consisting of a glassy carbon disk as the working electrode, a platinum wire as the auxiliary electrode and a Ag/AgCl system as the reference electrode. A 0.1 mol dm^-3^ solution of lithium perchlorate was used as supporting electrolyte and K_3_[Fe(CN)_6_] (*E*_1/2_ = 0,254 V *versus* Ag/AgCl; Δ*E* = 347 mV) was used as internal standard. The solutions were purged thoroughly with argon and kept under a positive pressure of this gas during the experiments. Scan rates were varied from 25 to 200 mV/s. Potentials are expressed *versus* NHE (*E*_1/2_(K_3_[Fe(CN)_6_]) = 0.361 V *vs* NHE) at 100 mV/s in aqueous solutions [22].

Gas chromatography analyses were conducted on a HP5890 gas chromatograph with a HPDB5 column (30 m × 0.25mm × 0.25 μm) connected to a FID detector, using H_2_ (140 kPa) as carrier gas. The analysis conditions for the cyclohexane oxidation reactions were: initial temperature of 50°C, heating ramp of 1.5°C /min to 56°C, then heating ramp of 10°C /min to the final temperature of 127°C. The injector and detector temperature were 200°C C and 250°C, respectively. Products were identified by their mass spectra and the retention times were compared with those of authentic samples. Quantification was made through calibration plots for the detector response of the authentic samples.

The catalase-like activities were followed by measuring the volume of O_2_ produced by H_2_O_2_ disproportionation reactions. The total reaction volume was kept constant during all experiments at 5.0 mL. The reactions were performed at 25°C, using the assistance of a water bath and a thermostat. TRIS buffer was used as solvent. The buffer pH was adjusted to 7.2 with HCl. The reactor was a kitassato flask (25 cm^3^) magnetically stirred and closed with a rubber septum. The kitassato was connected to an inverted graduate burette filled with water. Hydrogen peroxide solution (commercial 30% aqueous solution) was injected through the septum with a syringe and the dioxygen production was measured in the burette at appropriate times. The experimental data were plotted in a curve describing the amount of dioxygen evolved versus time.

**Caution!** The perchlorate salts used in this study are potentially explosive and should be handled with care!

### Syntheses

#### Synthesis of [Co(L)(H_2_O)](ClO_4_)_2_ (1)

[Co(L)(H_2_O)](ClO_4_)_2_ was synthesized by the addition of HL solution (3.0 mmol, 0.528 g in 10 cm^3^ of water) to a solution of Co(ClO_4_)_2_·6H_2_O (3.6 mmol, 1.317 g in 30 cm^3^ of methanol). After solvent evaporation, a red precipitate was formed. The solid was recrystallized from 1:4 methanol:ethyl acetate mixture. After few days, single crystals suitable for X-ray analysis were obtained. Yield: 0.82 g (49%).

*Anal*. Calc. for C_7_H_21_N_4_O_10_Cl_2_Co·H_2_O: C, 17.92; H, 4.94; N, 11.94. Found: C, 17.88; H, 5.03; N, 11.51. UV-Vis (H_2_O); λ/nm (ε/dm^3^ mol^-1^ cm^-1^): 366 (1.51×10^2^), 525 (6.42×10^1^). Ω (CH_3_CN) = 230 μS cm^-1^.

#### Synthesis of [Ni(HL)](ClO_4_)_2_ (2)

[Ni(HL)](ClO_4_)_2_ was synthesized by the addition of a HL solution (3.0 mmols, 0.528 g in 10 cm^3^ of water) to a solution of Ni(ClO_4_)_2_·6H_2_O (3.6 mmols, 1.314 g in 30 cm^3^ of methanol). After solvent evaporation, an orange precipitate was formed. The solid was recrystallized from 1:1, acetonitrile: cyclohexane mixture. After a few days, single crystals suitable for X-ray analysis were obtained. Yield: 0.58 g (37%).

*Anal*. Calc. for C_7_H_20_N_4_O_9_Cl_2_Ni: C, 19.38; H, 4.65; N, 12.91. Found: C, 19.84; H, 5.09; N, 12.68. UV-Vis (H_2_O); λ/nm (ε/dm^3^ mol^-1^ cm^-1^): 337 (sh), 442 (sh), 534 (5.74), 744 (4.22), 789 (4.09). Ω (CH_3_OH) = 158 μS cm^-1^.

#### Synthesis of [Cu(HL)](ClO_4_)_2_ (3)

Different authors have synthesized the complex **(3)** by adding HL to a methanolic solution of CuCl_2_ or Cu(N0_3_)_2_ followed by a column chromatography [19, 20]. Herein, (3) was synthesized by the addition of a HL solution (2.5 mmols, 0.44 g in 10 cm^3^ of water) to a solution of Cu(ClO_4_)_2_·6H_2_O (3.0 mmols, 1.11 g in 30 cm^3^ of water). The solvent was allowed to evaporate at 60 ^o^C and a purple precipitate was formed and washed with isopropyl alcohol. The solid was recrystallized in acetonitrile. Yield: 0.47g (36%).

*Anal*. Calc. for C_7_H_20_N_4_O_9_Cl_2_Cu: C, 19.16; H, 4.60; N, 12.77. Found: C, 19.42; H, 4.64; N, 12.35. UV-Vis (H_2_O); λ/nm (ε/dm^3^ mol^-1^ cm^-1^): 528 (7.52×10^1^). Ω (CH_3_OH) = 175.8 μS cm^-1^.

### X-ray diffraction experiments

The X-ray data for [Co(L)(H_2_O)](ClO_4_)_2_ (**1**) and Ni(HL)ClO_4_ (**2**) were collected from a *Bruker KAPPA CCD* diffractometer [23], using a selected single crystal at 295 K and *MoKα* monochromatic-graphite radiation. The cell parameters for the complexes were obtained using the *PHICHI* and *DIRAX* programs [24,25]. The average data were reduced using the *EvalCCD* program and the absorption correction was performed with the *SADABS* programs [26,27]. The structure was solved by direct methods via *SHELXS97* and refined via *SHELXL97* by a full-matrix least-squares treatment with anisotropic temperature parameters for all non H atoms [28].

For [Co(L)(H_2_O)](ClO_4_)_2_ the H atoms of the carbon and nitrogen were positioned geometrically (C–H = 0.97 Å for Csp^3^ atoms and N–H = 0.90 Å for Nsp^3^) and treated as riding on their respective methylene C atoms and amine N atoms, with *U*_iso_(H) values set at 1.2*U*_eq_Csp^3^ and 1.2*U*_eq_Nsp^3^. The positional parameters of the H atoms bonded to the oxygen atoms on the water molecules bonded in Cobalt atom in [Co(L)(H_2_O)](ClO_4_)_2_ were obtained from the difference Fourier map and refined with isotropic displacement parameters. The Flack parameter (0.77(2)) observed during the refinement of [Co(L)(H_2_O)](ClO_4_)_2_ indicated the configuration of the structure was inverted. After the inversion of the configuration, the new Flack parameter, 0.17(2), suggests the presence of racemic twin structures. In this way the crystalline refinement show an inversion twin, with orientation matrices assigned to the twin component, (-100, 0–10, 00–1) twin law. The finally refined ratio of the twin components shows a Flack parameter being 0.18(2): 0.82(2).

The crystal data are listed in Table 1 and in Supporting Information.

**Table 1:** Crystallographic data.

Compound	[Co(L)(H_2_O)](ClO_4_)_2_		[Ni(HL)](ClO_4_)_2_	
Empirical formula	C_14_H_40.67_Cl_4_Co_2_N_8_O_20_		C_7_H_20_Cl_2_N_4_NiO_9_	
Formula weight	902.22		433.86	
Temperature	293(2) K		293(2) K	
Wavelength	0.71073 Å		0.71073 Å	
Crystal system	Monoclinic		Monoclinic	
Space group	*Pc*		*P*21/*n*	
Unit cell dimensions	*a* = 10.334(2) Å	α = 90°.	*a* = 9.488(5)Å	α = 90°.
	*b* = 16.080(3) Å	β = 91.28(3)°.	*b* = 13.994(5) Å	β = 93.450(5)°
	*c* = 14.550(3) Å	γ = 90°.	*c* = 11.828(5) Å	γ = 90°.
Volume	2417.2(8) Å^3^		1567.6(12) Å^3^	
*Z*	3		4	
Density (calculated)	1.859 mg/m^3^		1.838 mg/m^3^	
Absorption coeficiente	1.455 mm^-1^		1.630 mm^-1^	
*F*(000)	1392		896	
Crystal size	0.29 × 0.20 × 0.03 mm^3^		0.062 × 0.200 × 0.212 mm^3^	
Theta range for data collection	2.75 to 25.49°		2.75 to 27.50°	
Index ranges	-11< = *h*< = 12, -19< = *k*< = 19, -16< = *l*< = 17		-12< = *h*< = 12, -18< = *k*< = 18, -15< = *l*< = 14	
Reflections collected	19276		19990	
Independent reflections	7331 [*R*(int) = 0.1238]		1908 [*R*(int) = 0.1143]	
Data / restraints / parameters	7331 / 2 / 654		3208 / 0 / 208	
Goodness-of-fit on *F*2	0.942		1.035	
Final *R* indices [*I*>2sigma(*I*)]	*R*_1_ = 0.0553, *wR*_2_ = 0.0831		*R*_1_ = 0.0541, *wR*_2_ = 0.0930	
*R* indices (all data)	*R*_1_ = 0.1435, *wR*_2_ = 0.1019		*R*_1_ = 0.1163, *wR*_2_ = 0.1104	
Absolute structure parameter	0.18(2)		*——*	
Largest diff. peak and hole	0.571 and -0.496 e.Å^-3^		0.406 and -0.403 e.Å^-3^	

### Reactivity studies

#### Catalase-like activity and kinetics of H_2_O_2_ dismutation

In order to study the kinetics of H_2_O_2_ dismutation, two sets of experiments were carried out in TRIS buffer solution at pH 7. In a first set of experiments, the initial concentration of H_2_O_2_ was kept constant (2.19 mol dm^-3^ for complex (1) and (3); 1.50 mol dm^-3^ for complex (2)) while the initial concentration of the catalyst was varied in the ranges indicated in Table 2. In a second set of experiments, the initial concentration of H_2_O_2_ was varied while the initial concentration of catalyst was kept constant and equal to 3.0 × 10^−3^ mol dm^-3^. Table 2 summarizes the H_2_O_2_ and catalyst concentrations values used in these experiments.

**Table 2:** Complexes and H_2_O_2_ concentration for the kinetics experiments at 25°C and pH 7.

Experiment	[H_2_O_2_] = fixed		[complex] = fixed	
	[H_2_O_2_]_0_/ mol dm^-3^	[complex]_0_/ mol dm^-3^	[H_2_O_2_]_0_/ mol dm^-3^	[complex]_0_/ mol dm^-3^
**[Co(L)(H**_**2**_ **O)](ClO**_**4**_ **)**_**2**_	2.19	2.19×10^−3^–1.09×10^−2^	0.60–4.20	3.0×10^−3^
**[Ni(HL)](ClO**_**4**_ **)**_**2**_	1.50	1.50×10^−3^–7.50×10^−3^	0.27–3.60	3.0×10^−3^
**[Cu(HL)](ClO**_**4**_ **)**_**2**_	2.19	2.19×10^−3^–1.09×10^−2^	0.6 to 4.20	3.0×10^−3^

The dioxygen evolution was measured volumetrically, the reactions were carried out at 25°C, using the assistance of a water bath and a thermostat. The total volume of the reaction solution was 5.0 mL. The reactor was a kitassato flask (25 cm^3^) magnetically stirred and closed with a rubber septum. The kitassato was connected to an inverted graduated burette filled with water. Hydrogen peroxide solution (commercial 30% aqueous solution) was injected through the septum with a syringe and the dioxygen production was measured in the burette at appropriate times. The experimental data were plotted in a curve describing the amount of dioxygen evolved *versus* time. Observed initial rates were expressed as mol(O_2_) s^-1^ and calculated from the maximum slope of the curves describing the O_2_ evolution *versus* time. All experiments were done, at least, in triplicate and the reported values are average values.

### Cyclohexane oxidation

Cyclohexane oxidation tests followed published procedure [29]. The reactions were carried out for 24 hours in CH_3_CN or H_2_O as solvent, at room temperature, under argon atmosphere, using H_2_O_2_ as oxidant and the complexes as catalysts. The catalyst:substrate:oxidant proportion used was 1:1000:1000, and the catalyst concentration was 7.0×10^−4^ mol dm^-3^. The reactions were quenched by the addition of an aqueous 0.4 M Na_2_SO_4_ solution, followed by extraction with 10 mL of diethyl ether. The organic layer was dried with anhydrous Na_2_SO_4_ and analyzed by gas chromatograph.

## Results and Discussion

### Syntheses

**Fig 1:**
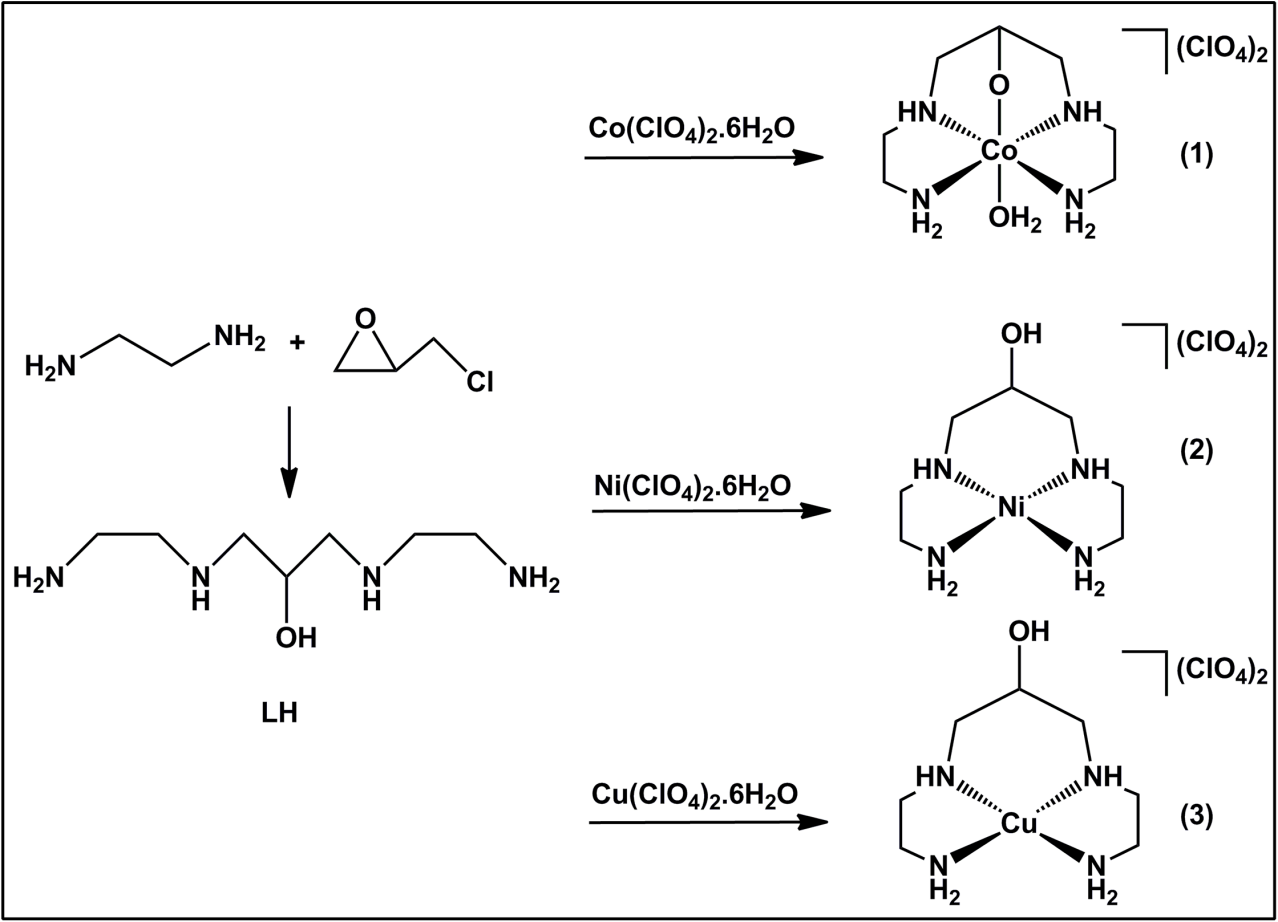
Syntheses of the complexes.

The ligand 1,3-bis[(2-aminoethyl)amino]-2-propanol was synthesized from epichlorohydrin and ethylenediamine, as described in literature [21]. The ligand has four aliphatics nitrogen atoms and one oxygen atom that can coordinate to the metallic center. The complexes were prepared by the addition of a HL solution to a solution of M(ClO_4_)_2_·6H_2_O (where M = Co, Ni or Cu) (Fig 1). The conductivity data of the three complexes are in agreement with a 2:1 electrolyte [30].

### X-ray crystallographic studies

Single crystals of the complexes [Co(L)(H_2_O)](ClO_4_)_2_ (**1**) and [Ni(HL)](ClO_4_)_2_ (**2**) were obtained and characterized by X-ray crystallography. The crystal structure of the complex [Cu(HL)](ClO_4_)_2_ (**3**) was reported previously in the literature [19,20], unit cell parameters of obtained single crystals were in accordance with the published data. The three complexes are mononuclear, cationic, with two perchlorate as counter-ions.

**Fig 2:**
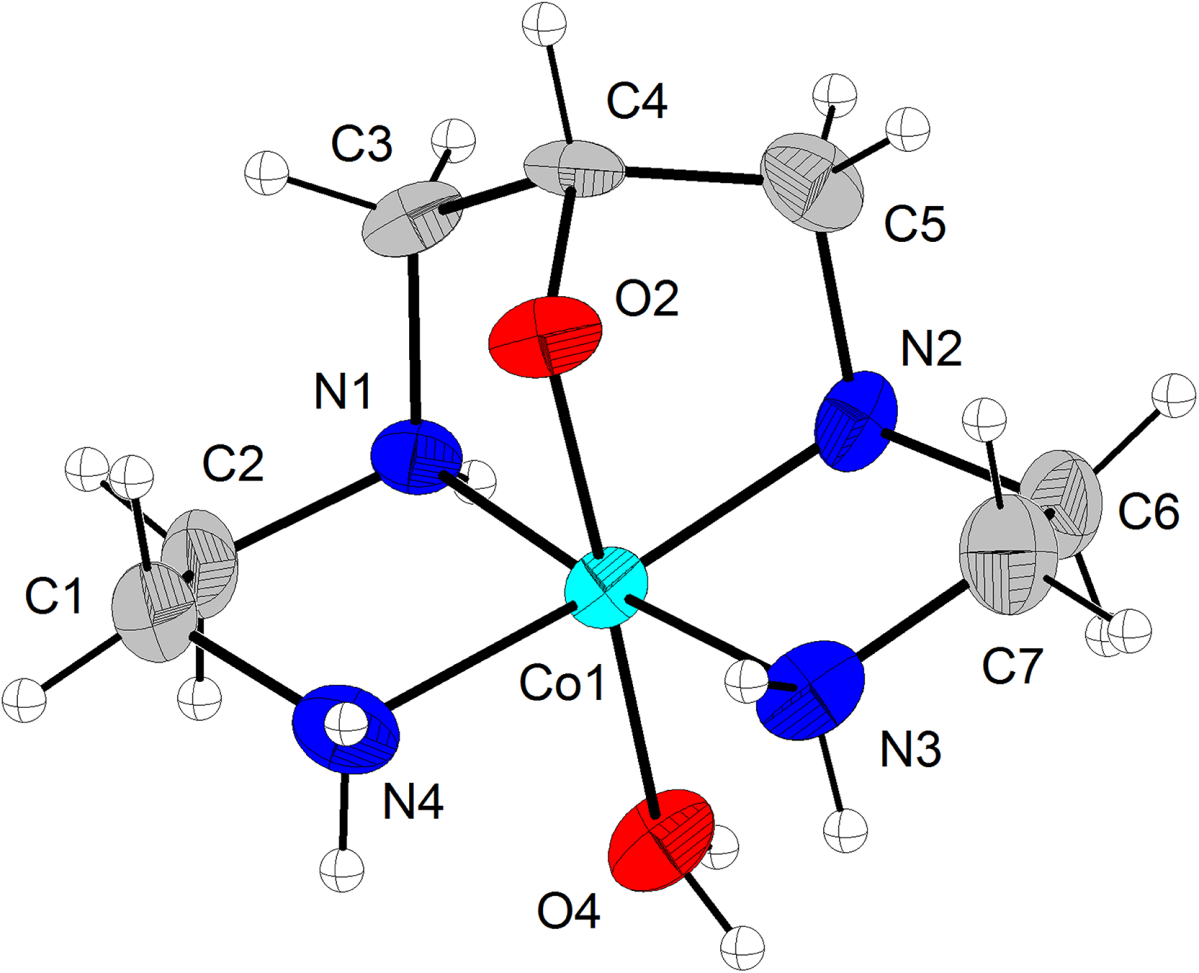
X-ray structure of [Co(L)(H_2_O)](ClO_4_)_2_. ORTEP plot and labeling scheme of [Co(L)(H_2_O)]^2+^, with thermal ellipsoid plot at 30% probability level.

**Fig 3:**
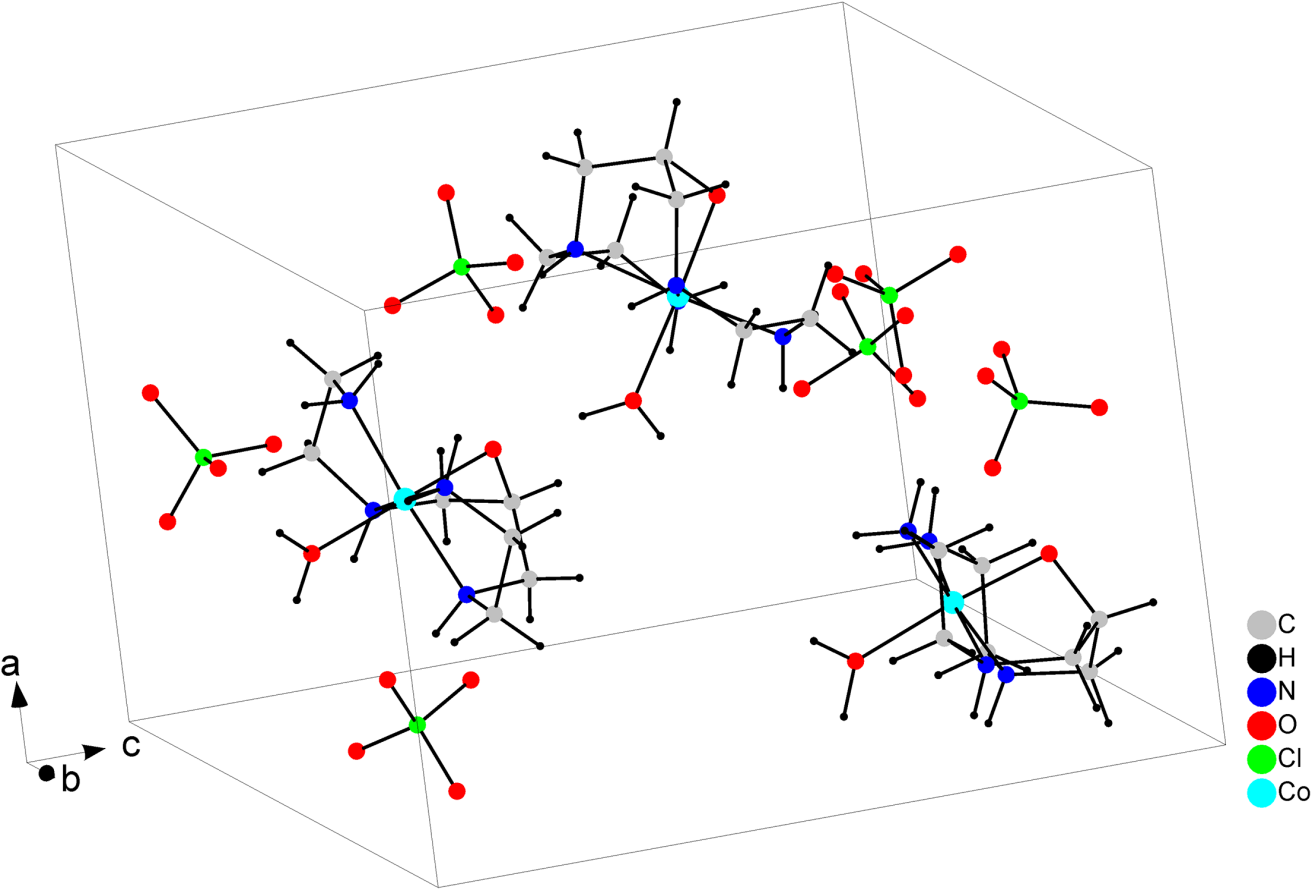
Unit cell arrangement of [Co(L)(H_2_O)](ClO_4_)_2_.

Fig 2 shows the ORTEP plot of [Co(L)(H_2_O)](ClO_4_)_2_, where the cobalt(III) ion adopts a distorted octahedral geometry by the coordination to the ligand and one water molecule. The ligand is coordinated by the four nitrogen atoms, which occupy the equatorial plane, as well as by the deprotonated alkoxo oxygen, which occupies the axial position *trans* to the water molecule. The unit cell (Fig 3) contains three crystallographic independent complex cations and six perchlorate anions. Table 3 presents the main bond distances and angles, Co-N and Co-O_H2O_ bonds are around 1.95 Å, while Co-O_alkoxo_ is smaller, 1.878 Å, due to the more basic character of the negatively charged group. [Co(L)(H_2_O)](ClO_4_)_2_ presented a high number of hydrogen bond interactions, basically between the N-H bonds of the ligand with the perchlorate ions (Table E in S2 File)).

**Table 3:** Selected bonds lengths (Å) and bond angles (°) for [Co(L)(H_2_O)](ClO_4_)_2_.

*Bond lengths*			
Co1-N1	1.963(7)	Co3-N10	1.947(8)
Co1-N2	1.975(8)	Co3-N11	1.957(6)
Co1-N3	1.970(7)	Co3-N12	1.976(7)
Co1-N4	1.952(7)	Co1-O2	1.874(6)
Co2-N5	1.962(8)	Co1-O4	1.979(6)
Co2-N6	1.959(8)	Co2-O6	1.866(5)
Co2-N7	1.956(7)	Co2-O8	1.983(6)
Co2-N8	1.966(8)	Co3-O10	1.883(5)
Co3-N9	1.968(8)		
*Bond angles*			
O2-Co1-N4	90.4(3)	N6-Co2-N8	177.1(4)
O2-Co1-N1	85.2(3)	N5-Co2-N8	87.2(3)
N4-Co1-N1	87.1(3)	O6-Co2-O8	173.9(3)
O2-Co1-N3	90.8(3)	N7-Co2-O8	91.4(3)
N4-Co1-N3	97.1(3)	N6-Co2-O8	88.7(3)
N1-Co1-N3	174.3(3)	N5-Co2-O8	91.5(3)
O2-Co1-N2	85.7(3)	N8-Co2-O8	93.4(3)
N4-Co1-N2	175.2(3)	O10-Co3-N10	85.7(3)
N1-Co1-N2	89.8(3)	O10-Co3-N11	90.0(3)
N3-Co1-N2	85.8(3)	N10-Co3-N11	87.1(3)
O2-Co1-O4	175.4(3)	O10-Co3-N9	85.5(3)
N4-Co1-O4	90.5(3)	N10-Co3-N9	90.4(3)
N1-Co1-O4	90.4(3)	N11-Co3-N9	174.9(3)
N3-Co1-O4	93.5(3)	O10-Co3-O12	176.9(3)
N2-Co1-O4	93.2(3)	N10-Co3-O12	91.3(3)
O6-Co2-N7	90.1(3)	N11-Co3-O12	90.7(3)
O6-Co2-N6	85.4(3)	N9-Co3-O12	93.7(3)
N7-Co2-N6	86.5(3)	O10-Co3-N12	90.6(3)
O6-Co2-N5	86.7(3)	N10-Co3-N12	175.9(3)
N7-Co2-N5	175.9(3)	N11-Co3-N12	94.8(3)
N6-Co2-N5	90.7(3)	N9-Co3-N12	87.5(3)
O6-Co2-N8	92.4(3)	O12-Co3-N12	92.3(3)
N7-Co2-N8	95.5(3)		

**Fig 4:**
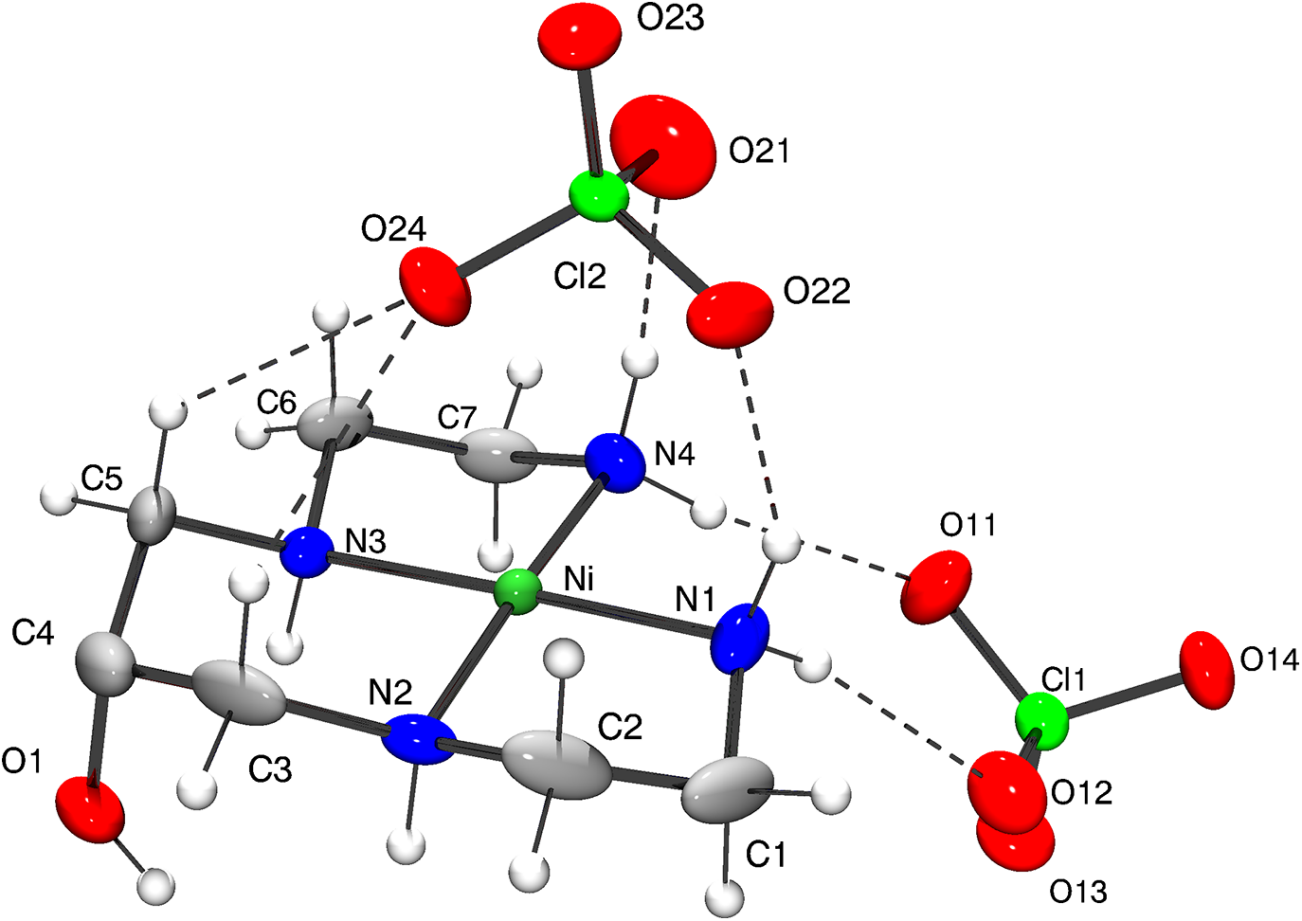
X-ray structure of [Ni(HL)](ClO_4_)_2_. ORTEP plot and labeling scheme of the asymmetric unit, with thermal ellipsoid plot at 30% probability level.

**Fig 5:**
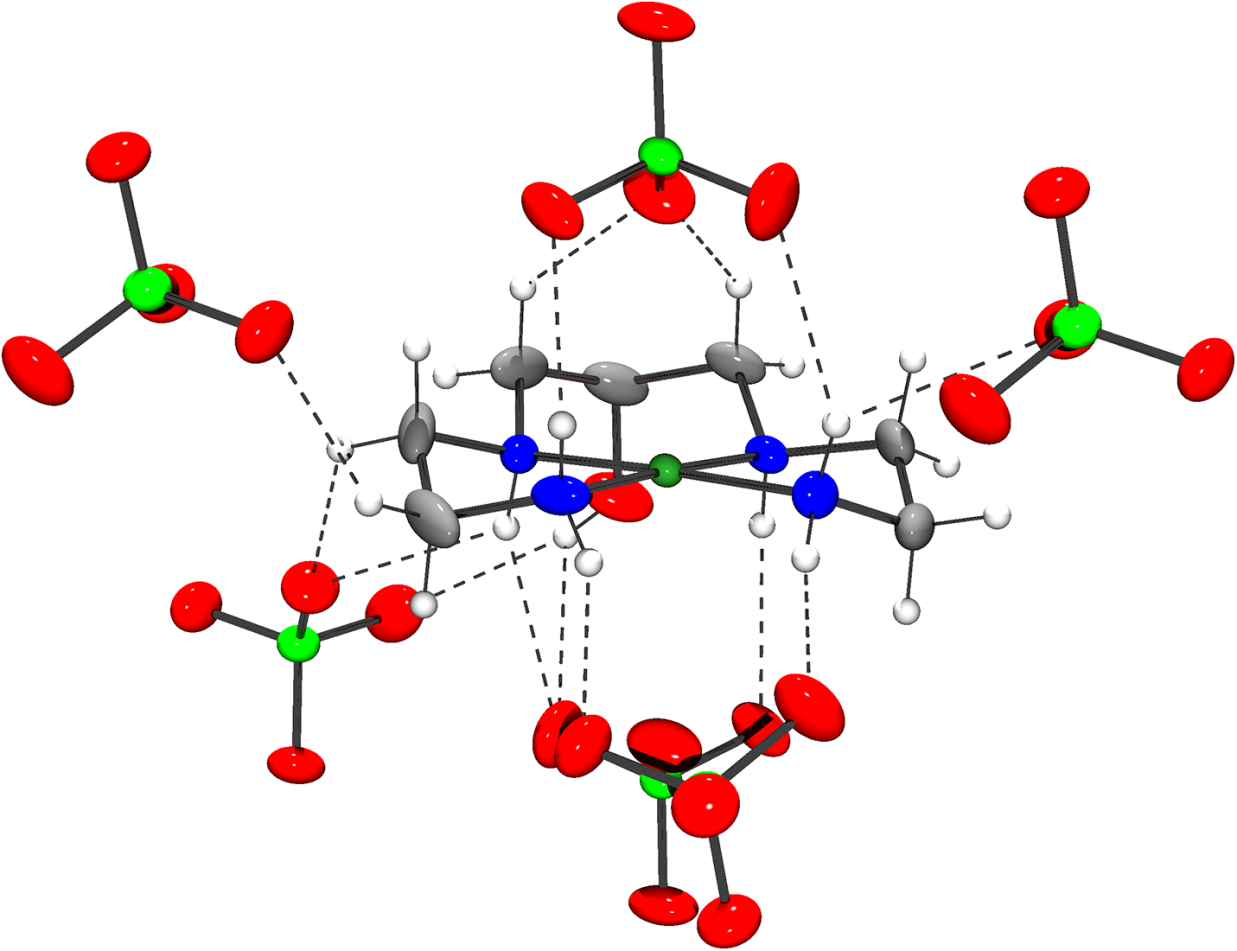
Intermolecular interactions between [Ni(HL)]^2+^ and ClO_4_^-^ units. Intermolecular interactions between the cationic unit and the neighbor perchlorate anions. Hydrogen bonds are represented as dashed lines.

The [Ni(HL)](ClO_4_)_2_ is a complex formed by a nickel(II) cationic unity [Ni(HL)]^2+^ and two perchlorate counter-ions, in order to keep the crystal neutral. The structure of the complex shows the square planar coordination of nickel (Figs 4 and 5). Table 4 presents selected bonds lengths and bond angles for [Ni(HL)](ClO_4_)_2_. The nickel and the nitrogen atoms are close to plane 1, through Ni and N1 to N4. Nickel is the most distant atom from this plane, with d[Ni–plane] = 0.032(2) Å. The nitrogen atoms are in the opposite side of the plane, if compared to nickel. The distances between the nitrogen atoms and the plane are in the interval between 0.007(2) Å and 0.009(2)Å. The C-C and C-N bond distances in the ligand are as expected for this type of compound. A similar structure was described for [Cu(HL)](ClO_4_)_2_ (**3**) [19,20], with Cu-N bond distances (around 2.0 Å) a bit longer than Ni-N bond distances in the herein discussed compound, which are close to 1.9 Å.

**Table 4:** Selected bonds lengths (Å) and bond angles (°) for [Ni(HL)](ClO_4_)_2_.

*Bond lengths*	
Ni-N1	1.907(5)
Ni-N2	1.915(4)
Ni-N3	1.915(4)
Ni-N4	1.903(4)
*Bond angles*	
N4-Ni-N1	92.0(2)
N1-Ni-N2	86.8(2)
N4-Ni-N3	87.18(19)
N2-Ni-N3	93.92(19)
N4-Ni-N2	177.40(19)
N1-Ni-N3	177.44(16)

The crystal of [Ni(LH)](ClO_4_)_2_ has two independent ClO_4_^-^ unities: perchlorate A containing atoms Cl1, O11, O12, O13 and O14; and perchlorate B containing atoms Cl2, O21, O22, O23 and O24. The nickel atom interacts, in opposite axial directions with two perchlorate B unities: oxygen O24 of one perchlorate interacts with the nickel atom, with d[Ni-O24] = 3.019 A, while oxygen O23i (symmetry operation i = -x+0.5, y+0.5,-z+0.5) of a second perchlorate is 2.988 Å far from Ni. Although these distances do not characterize a true chemical bond, it is important to emphasize (1) that these distances are smaller than the sum of van der Waals radii of Ni and O, and (2) that the angle O24—Ni—O23i (174.56°) is very close to 180° (Fig 5). Additionally, direction O24 –O23i is almost orthogonal to plane 1 (through Ni, N2, N3 and N4). In this way, these Ni–perchlorate interactions play important rule in the crystal packing. Perchlorate A, on the other hand, does not have any oxygen close to the metal.

It is also interesting to notice that the geometrical parameters of perchlorate anions A and B are similar. For anion A Cl-O bond distances are in the range from 1.416(4) to 1.437(6) A, with mean value of 1.424(3)A. For anion B, Cl–O bond distances range from 1.393(5) to 1.430(6) A, with mean value of 1.410(3) A. These values agree well to Cl–O previously reported bond distances of 1.414(26) A in ClO_4_^-^ [31]. O–Cl–O angles also suggest the two tetrahedral ClO_4_^-^ anions are similar: Perchlorate A has O–Cl–O bond angles ranging from 108.5(3)° to 110.7(3)°, while for perchlorate B these angles range from 107.9(4)° to 111.5(3)°.

As can be seen in Table E in S3 File, weak N—H…O hydrogen bonds, with d[N…O] around 3.0 A, and very weak C—H…O hydrogen bonds, with d[C…O] around 3.4 A and d[H…O]<2.7 A, contribute to the crystal packing as well. As observed by Taylor and Kennard [32] these interactions can be classified as hydrogen bonds. All of these interactions involve one cationic unit and one anionic perchlorate.

Therefore, the above discussion indicates that any intermolecular interactions (N—H…O, C—H…O or Ni…O) of the compound occur between one cationic unit and one counter-ion (Table E in S3 File, Fig 5), reflecting the importance of ClO_4_^-^ unities to the stabilization of the crystal structure.

### Infrared and electronic spectroscopy

The infrared spectra of the complexes (S1 Fig) presented the characteristic bands of the ligand with wavenumber shifts that indicates coordination to the metallic center. All three infrared spectra showed the stretches assigned to N-H and C-H of the aliphatic chain around 3200 and 2960 cm^-1^, respectively. The δ(N-H) and δ(C-H) were also observed around 1590 and 1460 cm^-1^. The spectra presented the Cl-O vibrations around 1100 and 625 cm^-1^, confirming the presence of perchlorate anions.

**Fig 6:**
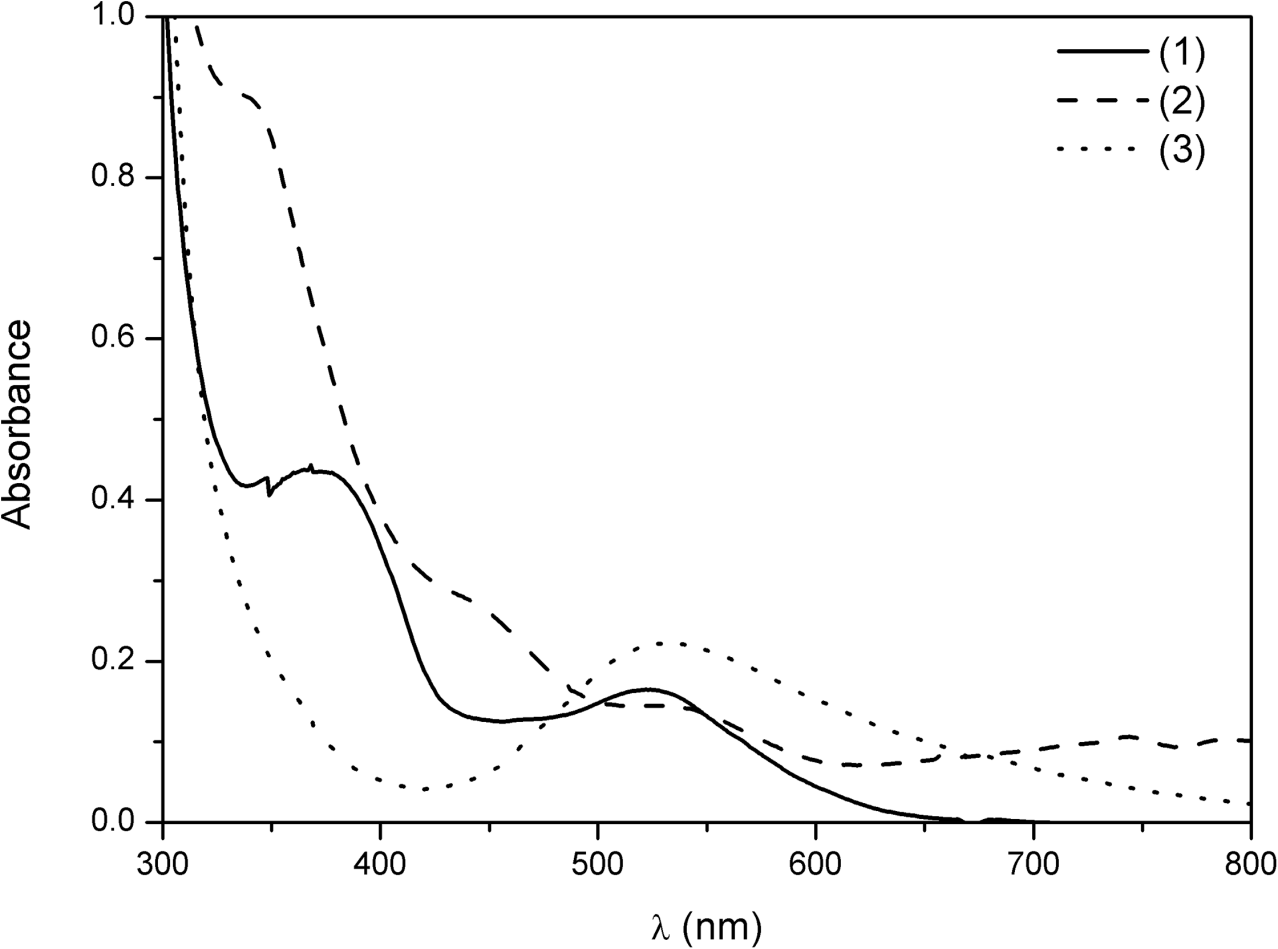
Electronic spectra of the complexes (1), (2), and (3) in water. [Co(L)(H_2_O)](ClO_4_)_2_ (**1**) and [Cu(HL)](ClO_4_)_2_ (**3**) at 3×10^−3^ mol dm^-3^, [Ni(HL)](ClO_4_)_2_ (**2**) at 3×10^−2^ mol dm^-3^.

The UV-Vis spectrum of (**1**) in aqueous solution (Fig 6) presented weak absorptions at 366 (ε = 1.51×10^2^ dm^3^ mol^-1^ cm^-1^) and 525 nm (ε = 6.42×10^1^ dm^3^ mol^-1^ cm^-1^), which may be attributed to the ligand field transitions ^1^A_1g_ → ^1^T_2g_ and ^1^A_1g_ → ^1^T_1g_, respectively [33]. Complex (**2**) spectrum (Fig 6) presented a higher number of low coefficient absorptivity bands, which is characteristic of d^8^ distorted octahedral geometry. According to Bussière and Reber, González and coworkers and Taran and coworkers, the bands at 744 (ε = 4.22×10^1^ dm^3^ mol^-1^ cm^-1^) and 789 nm (ε = 4.09×10^1^ dm^3^ mol^-1^ cm^-1^) may be attributed to the forbidden transition ^3^A_2g_ → ^1^E_g_ and to the allowed transition ^3^A_2g_ → ^3^T_1g_(F) [34–36]. However, it is not correct to ascribe each one since there is spin orbit coupling between them [37]. In turn, the band at 337 nm (ε = 3.22×10^1^ dm^3^ mol^-1^ cm^-1^) may be attributed to the high energy transition ^3^A_2g_ → ^3^T_1g_(P) while the band at 534 nm (ε = 5.74×10^1^ dm^3^ mol^-1^ cm^-1^) may be related to the forbidden transition ^3^A_2g_ → ^1^T_2g_. The bands at 442 nm (ε = 1.16×10^1^ dm^3^ mol^-1^ cm^-1^) are related to the split of ^3^T_1g_ (P). The spectrum of (**3**) (Fig 6) showed only a ligand field transition at 528 nm (ε = 7.52×10^1^ dm^3^ mol^-1^ cm^-1^), which may be attributed to the ^2^E_g_ → ^2^T_2g_. The electronic spectra are characteristic of distorted octahedral geometry of a d^6^ low-spin (**1**), d^8^ (**2**) and d^9^ systems (**3**), respectively, what suggested the coordination of the Cu(II) and Ni(II) complexes to water to complete the six coordination positions [33,37].

### ESI-MS

The ESI-MS spectra of the complexes in methanol (Figures A-C in S1 File) showed only few peaks and, apparently, the ligand did not deteriorate in solution, remaining coordinated to the metals. The peaks at *m/z* 145.001, *m/z* 279.102, and *m/z* 363.155 observed in the spectrum of [Co(L)(H_2_O)](ClO_4_)_2_ can be related to the association of the complex with solvent (methanol) and N_2_ molecules present in the gas phase, tentatively ascribed to ions with *z* equal 2 and 1, [Co^III^(L)(N_2_)_2_]^2+^, [Co^III^(L-H^+^)(H_2_O)(N_2_)]^1+^, and [Co^III^(L-(NH_3_)_2_)(MeOH)_2_(ClO_4_)]^1+^, respectively [38]. The peak at *m/z* 163.057 does not belong to the complex and is due to the calibration mixture.

The ESI-MS spectrum of [Ni(HL)](ClO_4_)_2_ allowed the observation of ions with *z* equal 2, [Ni^II^(HL)]^2+^, [Ni^II^(HL)(N_2_)_2_]^2+^, and [Ni^II^(HL)(MeOH)_3_(N_2_)]^2+^ assigned to *m/z* 117.014, *m/z* 144.951, and *m/z* 178.977, respectively. The peak at *m/z* 233.046 can be attributed to the ion with *z* equal 1, [Ni^II^(L)]^+^.

For [Cu(HL)](ClO_4_)_2_ the peaks at *m/z* 238.0844 and *m/z* 338.0386 were assigned to the ions with *z* equal 1, [Cu^II^(L)]^+^ and [Cu^II^(L)(ClO_4_)]^+^, respectively.

### Cyclic Voltammetry

**Fig 7:**
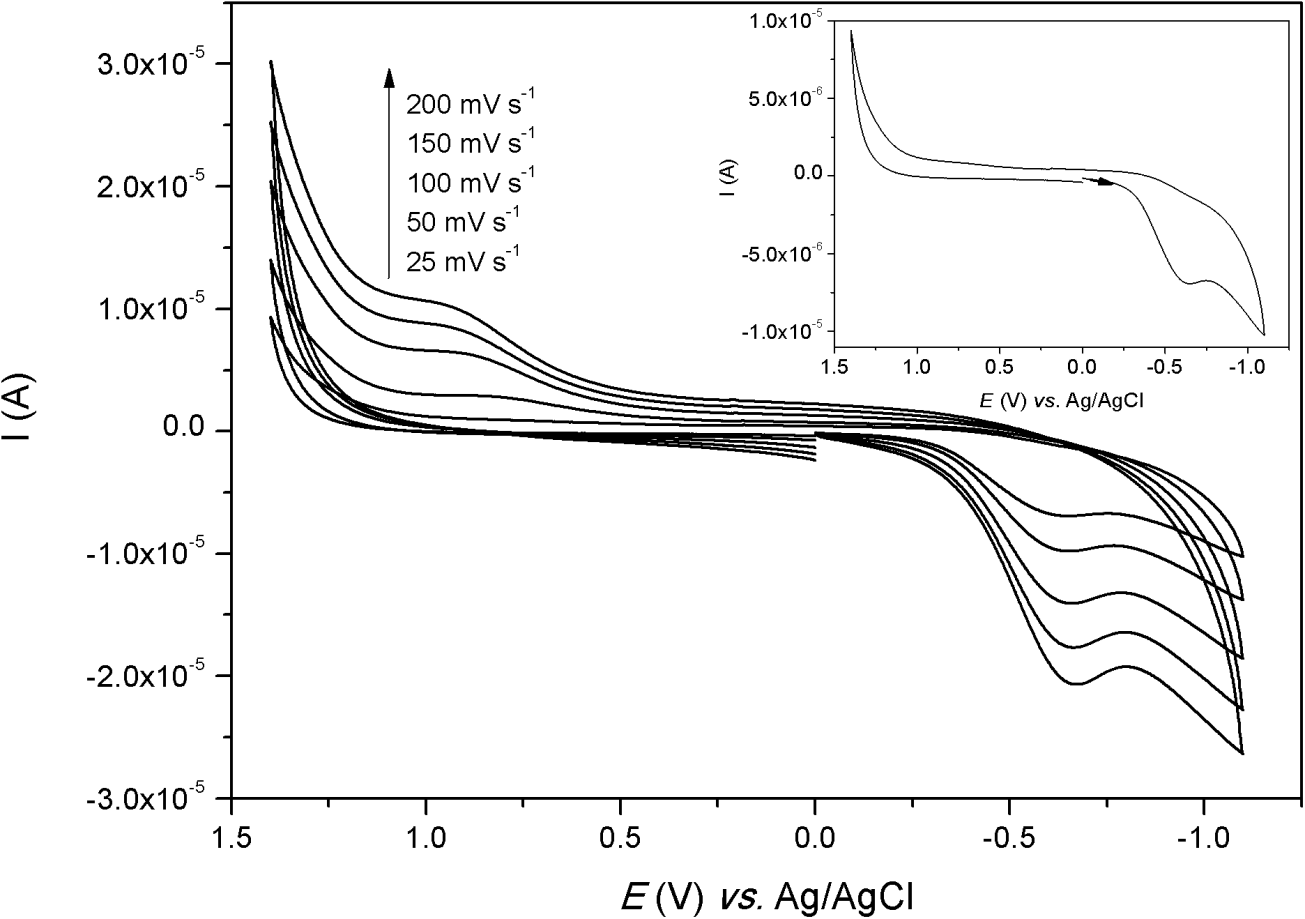
Cyclic voltammogram of [Co(L)(H_2_O)](ClO_4_)_2_, in water. K_3_[Fe(CN)_6_] (*E*_1/2_ = 0,254 V *versus* Ag/AgCl; Δ*E* = 347 mV) was used as internal standard. Inlet: voltammograms at 25 mV s^-1^.

**Fig 8:**
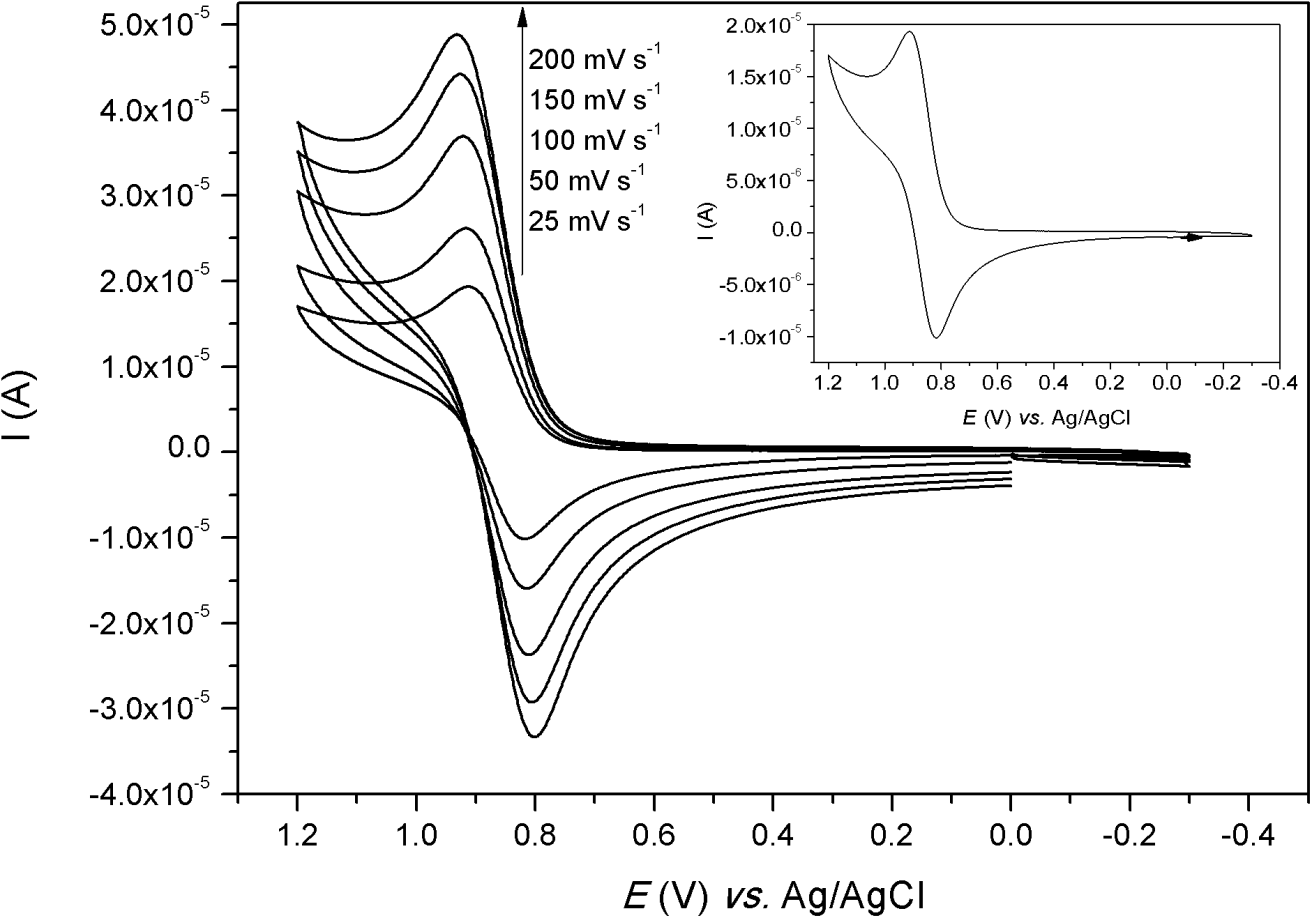
Cyclic voltammogram of [Ni(HL)](ClO_4_)_2_, in water. K_3_[Fe(CN)_6_] (*E*_1/2_ = 0,254 V *versus* Ag/AgCl; Δ*E* = 347 mV) was used as internal standard. Inlet: voltammograms at 25 mV s^-1^.

**Fig 9:**
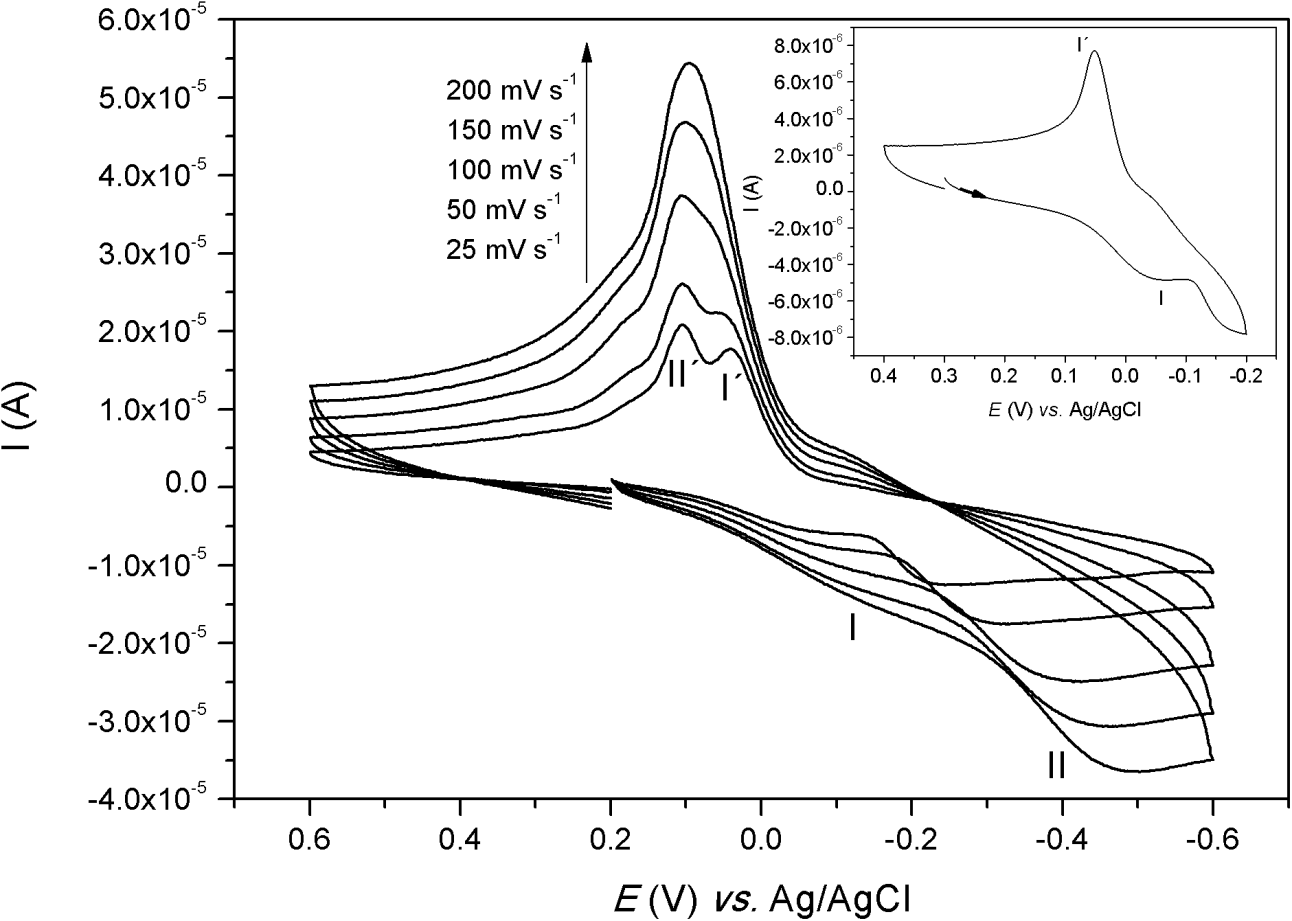
Cyclic voltammogram of [Cu(HL)](ClO_4_)_2_, in water. K_3_[Fe(CN)_6_] (*E*_1/2_ = 0,254 V *versus* Ag/AgCl; Δ*E* = 347 mV) was used as internal standard. Inlet: voltammograms at 25 mV s^-1^.

The electrochemical behavior of the complexes was investigated in water (Figs 7–9). The voltammograms of complexes [Co(L)(H_2_O)](ClO_4_)_2_ and [Ni(HL)](ClO_4_)_2_ showed only one process and the redox potential values are presented in Table 5. For complex [Co(L)(H_2_O)](ClO_4_)_2_, the cathodic wave was observed at -0.641 V *vs*. Ag/AgCl (-0.432 V *vs*. NHE) and the anodic wave was observed at 0.837 V *vs*. Ag/AgCl (1.046 V *vs*. NHE). This can be considered an irreversible process, which is a typical electrochemical behavior for cobalt complexes due to spin shifts from low spin Co(III) to high spin Co(II). The reduction wave can be assigned to the one-electron reduction process from Co(III) → Co(II) and the anodic wave can be assigned to the reverse process [39]. The voltammogram of [Ni(HL)](ClO_4_)_2_ presented only one process with the anodic wave at 0.920 V *vs*. Ag/AgCl (1.13 V *vs*. NHE) and the cathodic wave at 0.812 V *vs*. Ag/AgCl (1.02 V *vs*. NHE). Although the Δ*E* (109 mV) value is smaller than the Δ*E* (347 mV) found for the standard K_3_[Fe(CN)_6_], this process can be considered as a quasi-reversible system since *i*_pa_/*i*_pc_ (1.28) is different from one and Δ*E* changes with the scan rates. This process may be assigned to the oxidation of Ni(II) to Ni(III) and the reverse scan to the reduction of Ni(III) to Ni(II) [40].

**Table 5:** Cyclic voltammetry data for the complexes.

	[Co(L)(H_2_O)](ClO_4_)_2_	[Ni(HL)](ClO_4_)_2_	[Cu(HL)](ClO_4_)_2_
**E**_**pc**_ **(V) vs. Ag/AgCl**	-0.641	0.812	- 0.032 (I)
			-0.198 − -0.450 (II)
**E**_**pa**_ **(V) vs. Ag/AgCl**	0.837	0.920	0.049 (I`)
			0.103 (II´)
**E**_**1/2**_ **(V) vs. Ag/AgCl**	-	0.866	0.009 (I and I´)
**E**_**1/2**_ **(V) vs. NHE**	-0.432ª	1.08	0.218 (I and I´)
	1.05^b^		
**ΔE(V)**	-	0.109	0.081
**ΔE(V)**_**internal standard**_	0.347	0.347	0.347

^a^: *E*_pc_ is given instead of *E*_1/2_

^b^: *E*_pa_ is given instead of *E*_1/2_.

The electrochemical behavior of complex [Cu(HL)](ClO_4_)_2_ was different from the other complexes, when scanning over -0.2 V, the voltammogram presented two cathodic and two anodic waves. But when the scan was reversed prior to the second reduction, only one process could be observed, as showed in the inlet of Fig 9. This process presented the cathodic peak at -0.032 V *vs*. Ag/AgCl (0.177 V *vs*. NHE—I) and the anodic peak at 0.049 V *vs*. Ag/AgCl (0.258 V *vs*. NHE—I´), which can be attributed to the redox couple Cu(II)-Cu(I). Scanning over -0.2 V, another cathodic peak (II) was observed. This peak shifted with the scan rate and can be tentatively attributed to the reduction of Cu(I) to metal copper. The anodic wave (II´) at 0.104 V *vs*. Ag/AgCl (0.313 V *vs*. NHE) might be assigned as the redissolution of the metal copper formed previously [41,42]. The electrochemical equilibrium between processes I and I´ may be very slow, since at fast scan rates the anodic peak I´ disappeared. Alternatively, process II´ may involve so much current that occulted process I´.

### Reactivity in the presence of H_2_O_2_

The three mononuclear complexes were tested as catalysts for the dismutation of H_2_O_2_ and for the oxidation of cyclohexane, using H_2_O_2_ as oxidant. Neither of the complexes presented satisfactory yields in the formation of cyclohexanol and cyclohexanone (S1 Table), but the three complexes exhibited catalase-like activity under the studied conditions. The oxidations yields and the kinetic parameters are summarized in Table 6. Control experiments with Co(ClO_4_)_2_, Ni(ClO_4_)_2_ and Cu(ClO_4_)_2_ at 3.0×10^−3^ M and H_2_O_2_ 3.0 M showed no hydrogen peroxide decomposition.

**Table 6:** Reactivity results in presence of H_2_O_2_.

Complex	Catalase-like activity		
	*K*_M_ (mol dm^-3^)	*k*_cat_ (s^-1^)	*k*_*cat*_/*K*_*M*_ (mol^-1^ dm^3^ s^-1^)
(**1**)^*a*^	3.08	5.10×10^−5^	1.67×10^−5^
(**2**)^*a*^	2.75	2.60×10^−4^	9.45×10^−5^
(**3**)^*a*^	17.4	1.54×10^−3^	8.87×10^−5^
*T*. *thermophiles* [36]	0.083 (0.008)	2.6×10^5^	3.13×10^6^
*T*. *álbum* [36]	0.015	2.6×10^4^	1.73×10^6^
*L*. *plantarum* [36]	0.35	2×10^5^	0.57×10^6^
[Cu(*N*-baa)_2_(phen)]^b^ ^,^ ^c^ [14]	52×10^−3^	6.62×10^−2^	1.27
[Fe_4_(μ-O)(μ-OH)(μOAc)_4_(L)_2_](ClO_4_)_3_ ^a^	2.882	3.50×10^−3^	1.21×10^−3^

^*a*^ catalase-like activity measured in water (pH 7)

^*b*^ catalase-like activity measured in DMF

^*c*^ *N*-baaH: *N*-benzoylanthranilic acid, phen: 1,10-phenanthroline

The H_2_O_2_ decomposition was followed measuring the dioxygen evolution during the reaction. The initial rates method was applied in order to determine the kinetic parameters. The initial rate values were calculated as the maximum slope of the curves of mol(O_2_) *versus* time. S2 Fig shows the dioxygen evolution as function of time for the three complexes.

The plots of the logarithm of the initial rates (*v*_0_) *versus* logarithm of complex concentration (S3 Fig) for the three complexes exhibited a linear dependence with the catalysts. The slopes found were 0.97 ± 0.07 for complex (**1**) (R = 0.97), 0.97 ± 0.03 for complex (**2**) (R = 0.99) and 0.99 ± 0.06 for complex (**3**) (R = 0.98), indicating in all cases a first-order reaction in relation to the complex.

**Fig 10:**
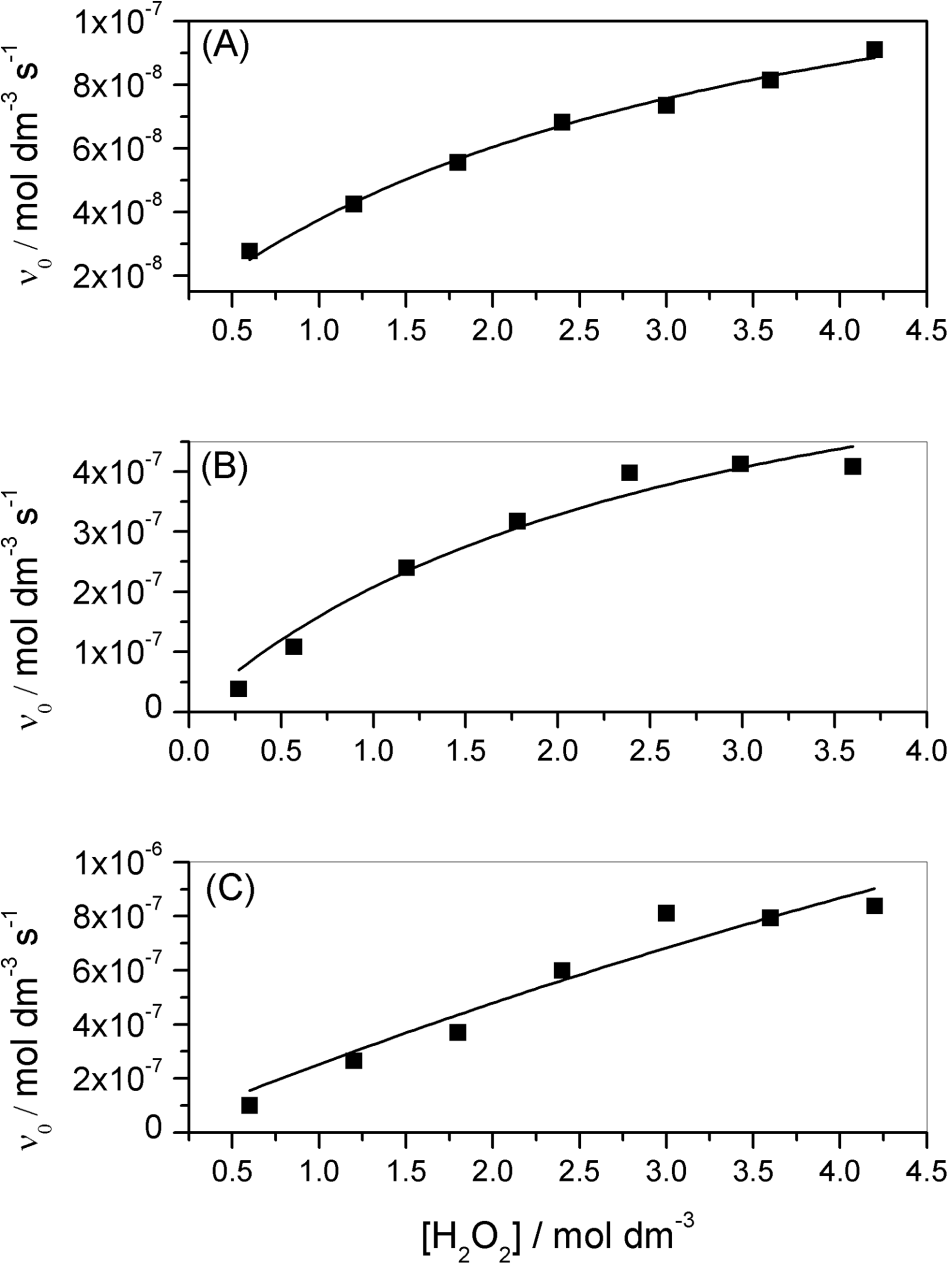
Initial rate (*v*_0_) of O_2_ evolution as a function of initial H_2_O_2_ concentration. Experimental data (square); Nonlinear Least Square fitting using Michaelis-Menten equation (continuous line). (A) [Co(L)(H_2_O)](ClO_4_)_2_; (B) [Ni(HL)](ClO_4_)_2_; (C) [Cu(HL)](ClO_4_)_2_. [complex] = 3.0×10^−3^ mol dm^-3^.

The plots of initial rates *versus* hydrogen peroxide concentration showed saturation kinetics in relation to the substrate, indicating a Michaelis-Menten catalytic behavior (Eq 2), similar to the natural catalase enzymes. Besides that, the solvent used in the experiments was water, which is closer to the medium of the enzymatic reactions and consequently provides more accurate data for comparison. The saturation graphics are presented in Fig 10.

$$v_{0}=\frac{k_{cat}\left[complex\right]\left[H_{2}O_{2}\right]}{K_{M}+\left[H_{2}O_{2}\right]}$$ (2)

To calculate the Michaelis-Menten parameters (Table 6), the more reliable nonlinear least square fit was used instead of a linear fit, following the recommendation of different authors [43,44]. [Ni(HL)](ClO_4_)_2_ (**2**) presented the higher affinity to the substrate (1/*K*_M_), followed closely by complex [Co(L)(H_2_O)](ClO_4_)_2_ (**1**), but [Cu(HL)](ClO_4_)_2_ (**3**) presented a very low affinity, with *K*_M_ value roughly five-times higher in relation to the other complexes. Complex (**2**) also presented the best catalytic efficiency (*k*_cat_/*K*_M_) between the three complexes, however, it was still very low if compared with the natural enzymes [45].

A few number of copper and cobalt complexes has been described as catalase synthetic model, but most of them were studied in organic solvents, such as DMF and acetonitrile [11–14,16,17], or have presented significant activity only over pH 8 [7,8]. Furthermore, none of these complexes have presented the Michaelis-Menten behavior, except the complex [Cu(*N*-baa)_2_(phen)] (where *N*-baaH is *N*-benzoylanthranilic acid and phen is 1,10-phenanthroline), which had its kinetic studies carried out in DMF [14], and the complex [Fe_4_(μ-O)(μ-OH)(μ-OAc)_4_(L)_2_](ClO_4_)_3_ (HL is the ligand 1,3-bis[(2-aminoethyl)amino]-2-propanol) [46], which was studied in the same conditions of the present work. Complex (**2**) presented catalytic activity (*k*_cat_/*K*_M_) ten-fold lower than the tetranuclear iron complex [Fe_4_(μ-O)(μ-OH)(μ-OAc)_4_(L)_2_](ClO_4_)_3_, which has the same ligand. However, complex (**2**) is a mononuclear complex while [Fe_4_(μ-O)(μ-OH)(μ-OAc)_4_(L)_2_](ClO_4_)_3_ presented four iron centers. So, for a more fair comparison, the initial rate of (**2**) at a concentration (2.5×10^−3^ mol dm^-3^) roughly four times higher than [Fe_4_(μ-O)(μ-OH)(μ-OAc)_4_(L)_2_](ClO_4_)_3_ (5.8×10^−4^ mol dm^-3^) was compared, and (**2**) presented initial rate (2.0×10^−7^ mol dm^-3^ s^-1^) around 1.7 times higher than the tetranuclear complex (1.2×10^−7^ mol dm^-3^ s^-1^).

Actually, there are few catalase-like activity studies in water because they are usually hampered by the low solubility of most of the complexes or even by the low activity in aqueous solution. In this sense, the results described herein are promising when compared with the previously reported because it shows the activity in a medium similar to that where the natural enzymes work, *i*.*e*., aqueous solution and physiological pH. Additionally, as far as we know, complex (**2**) was the first nickel complex reported presenting a significant catalase-like activity. Siegel at al. reported that the complex [Ni(en)_3_]^2+^, has a rate constant, *k*, equal to 3.5×10^−11^ dm^3^ mol^-1^ s^-1^, which is lower than the catalase activity of Ni^2+^(aq) (*k* = 1.77×10^−9^ dm^3^ mol^-1^ s^-1^) [47]. These authors have also studied Ni(II) complexes with 2,2'-bipyridyl, 2-picolylamine, 4-aminomethylimidazole, and histamine. The experimental data for the first complex (see Figure 5 in the Siegel at al. article [47]) show that the catalase activity follows [Ni^2+^(aq)] and the other complex do not shown catalase activity at all. Then, the relatively high activity promoted by complex **2** should encourage additional studies on nickel synthetic models for catalase.

## Conclusions

Three mononuclear complexes were synthesized, characterized and their catalase-like activity were investigated. The kinetic studies were conducted in a very similar condition to the biological enzymes, *i*.*e*., aqueous solution and neutral pH. All complexes obeyed Michaelis-Menten kinetics and the following order of activity was found: [Ni(HL)](ClO_4_)_2_ (**2**) > [Cu(HL)](ClO_4_)_2_ (**3**) > [Co(L)(H_2_O)](ClO_4_)_2_ (**1**). The most active complex is also the first example of a nickel compound presenting a significant catalase-like activity, which can propel new studies on nickel complex synthetic models.

CCDC 990939 and CCDC 990479 contain the supplementary crystallographic data for complex [Co(L)(H_2_O)](ClO_4_)_2_ (**1**) and [Ni(HL)](ClO_4_)_2_ (**2**), respectively. These data can be obtained free of charge from The Cambridge Crystallographic Data Centre via www.ccdc.cam.ac.uk/data_request/cif.

## Supporting Information

S1 Fig Infrared spectra of complexes. [Co(L)(H_2_O)](ClO4)_2_ (1), [Ni(HL)](ClO_4_)_2_ (2) and [Cu(L)](ClO_4_)_2_ (3).
(DOCX)

S2 Fig Evolution of the O_2_ from H_2_O_2_ dismutation catalized by complexes (1), (2), and (3) in TRIS buffer at pH 7.2. (DOCX)

S3 Fig Plots of the logarithm of the initial rate (ν_0_) as a function of the logarithm of complex concentration. (DOCX)

S1 File ESI-MS spectrum of complexes [Co(L)(H_2_O)](ClO_4_)_2_ (Figure A), [Ni(HL)](ClO_4_)_2_ (Figure B), [Cu(HL)](ClO_4_)_2_ (Figure C), and isotopic profile simulation (Figure D) of m/z 117.014 of the complex [Ni(HL)](ClO_4_)_2_ in methanol. (DOCX)

S2 File Crystal data and structure refinement (Table A), atomic coordinates, equivalent isotropic displacement parameters (Table B), bond lengths, angles (Table C), anisotropic displacement parameters (Table D), and selected intermolecular interactions parameters (Table E) for [Co(L)(H_2_O)](ClO_4_)_2_ (1) (DOCX)

S3 File Crystal data and structure refinement (Table A), atomic coordinates, equivalent isotropic displacement parameters (Table B), bond lengths, angles (Table C), anisotropic displacement parameters (Table D), and selected intermolecular interactions parameters (Table E) for [Ni(L)](ClO_4_)_2_ (2). (DOCX)

S1 Table Yield of cyclohexanol and cyclohexanone after 24 hours in CH_3_CN or H_2_O as solvent. (DOCX)
