# Supplementary material for: Synthesis and Characterization of Cobalt(III), Nickel(II) and Copper(II) Mononuclear Complexes with the Ligand 1,3-bis[(2-aminoethyl)amino]-2-propanol and Their Catalase-Like Activity
Source: PLoS One. 2015 Sep 17;10(9):e0137926. doi: 10.1371/journal.pone.0137926 (PMC4574563; doi:10.1371/journal.pone.0137926)
Supplement: S1 Fig — [Co(L)(H2O)](ClO4)2 (1), [Ni(HL)](ClO4)2 (2) and [Cu(L)](ClO4)2 (3). (DOCX) [file pone.0137926.s001.docx]

Supporting Information for

#### Synthesis and Characterization of Cobalt(III), Nickel(II) and Copper(II) Mononuclear Complexes with the Ligand 1,3-bis[(2-aminoethyl)amino]-2-propanol and their Catalase-like Activity.

Bianca M. Pires^1¶^, Daniel M. Silva^1^, Lorenzo C. Visentin^2&^, Bernardo L. Rodrigues^3&^, Nakédia M. F. Carvalho^1¶,#a^, Roberto B. Faria^1¶*^

^1^ Instituto de Química, Universidade Federal do Rio de Janeiro, Rio de Janeiro, Rio de Janeiro, Brazil

^2^ NanoBusiness Informação e Inovação Ltda., Rio de Janeiro, Rio de Janeiro, Brazil

^3^ Departamento de Química, Universidade Federal de Minas Gerais, Belo Horizonte, Minas Gerais, Brazil

^#a^ Current Address: Instituto de Química, Universidade do Estado do Rio de Janeiro, Rio de Janeiro, Rio de Janeiro, Brazil

* Corresponding author

E-mail: [faria@iq.ufrj.br](mailto:faria@iq.ufrj.br) (RBF)

**S1 Figure.** Infrared spectra of complexes [Co(L)(H_2_O)](ClO_4_)_2_ (**1**), [Ni(HL)](ClO_4_)_2_ (**2**) and [Cu(L)](ClO_4_)_2_ (**3**).
