# Supplementary material for: Synthesis and Characterization of Cobalt(III), Nickel(II) and Copper(II) Mononuclear Complexes with the Ligand 1,3-bis[(2-aminoethyl)amino]-2-propanol and Their Catalase-Like Activity
Source: PLoS One. 2015 Sep 17;10(9):e0137926. doi: 10.1371/journal.pone.0137926 (PMC4574563; doi:10.1371/journal.pone.0137926)
Supplement: S2 File — (DOCX) [file pone.0137926.s005.docx]

Supporting Information for

#### Synthesis and Characterization of Cobalt(III), Nickel(II) and Copper(II) Mononuclear Complexes with the Ligand 1,3-bis[(2-aminoethyl)amino]-2-propanol and their Catalase-like Activity.

Bianca M. Pires^1¶^, Daniel M. Silva^1^, Lorenzo C. Visentin^2&^, Bernardo L. Rodrigues^3&^, Nakédia M. F. Carvalho^1¶,#a^, Roberto B. Faria^1¶*^

^1^ Instituto de Química, Universidade Federal do Rio de Janeiro, Rio de Janeiro, Rio de Janeiro, Brazil

^2^ NanoBusiness Informação e Inovação Ltda., Rio de Janeiro, Rio de Janeiro, Brazil

^3^ Departamento de Química, Universidade Federal de Minas Gerais, Belo Horizonte, Minas Gerais, Brazil

^#a^ Current Address: Instituto de Química, Universidade do Estado do Rio de Janeiro, Rio de Janeiro, Rio de Janeiro, Brazil

* Corresponding author

E-mail: [faria@iq.ufrj.br](mailto:faria@iq.ufrj.br) (RBF)

**Table A.** Crystal data and structure refinement for [Co(L)(H_2_O)](ClO_4_)_2_ (**1**).

Identification code complex 1_[CoL(ClO4)2]

Empirical formula C14 H40.67 Cl4 Co2 N8 O20

Formula weight 902.22

Temperature 293(2) K

Wavelength 0.71073 Å

Crystal system monoclinic

Space group *Pc*

Unit cell dimensions a = 10.334(2) Å a= 90°.

b = 16.080(3) Å b= 91.28(3)°.

c = 14.550(3) Å g = 90°.

Volume 2417.2(8) Å3

Z 3

Density (calculated) 1.859 Mg/m3

Absorption coefficient 1.455 mm-1

F(000) 1392

Crystal size 0.29 x 0.20 x 0.03 mm3

Theta range for data collection 2.75 to 25.49°.

Index ranges -11<=h<=12, -19<=k<=19, -16<=l<=17

Reflections collected 19276

Independent reflections 7331 [R(int) = 0.1238]

Completeness to theta = 25.49° 89.9 %

Max. and min. transmission 0.9576 and 0.6776

Refinement method Full-matrix least-squares on F2

Data / restraints / parameters 7331 / 2 / 654

Goodness-of-fit on F2 0.942

Final R indices [I>2sigma(I)] R1 = 0.0553, wR2 = 0.0831

R indices (all data) R1 = 0.1435, wR2 = 0.1019

Absolute structure parameter 0.18(2)

Largest diff. peak and hole 0.571 and -0.496 e.Å-3

**Table B.** Atomic coordinates (× 104) and equivalent isotropic displacement parameters (Å2 × 103) for [Co(L)(H_2_O)](ClO_4_)_2_ (**1**). U(eq) is defined as one third of the trace of the orthogonalized Uij tensor.

________________________________________________________________________________

x y z U(eq)

________________________________________________________________________________

C(1) 2199(9) 5779(6) 8766(7) 43(3)

C(2) 798(10) 5998(6) 8616(7) 39(3)

C(3) 681(8) 7394(5) 9354(5) 29(2)

C(4) 1777(8) 8037(5) 9331(6) 27(2)

C(5) 1486(9) 8762(6) 8685(6) 38(3)

C(6) 2260(9) 8901(6) 7069(7) 39(3)

C(7) 3671(9) 8658(6) 7226(7) 44(3)

C(8) 8745(11) 5990(6) 4642(7) 57(4)

C(9) 8945(12) 6294(6) 3678(7) 53(3)

C(10) 10746(9) 7338(6) 3913(6) 41(3)

C(11) 10907(9) 7935(7) 4701(7) 45(3)

C(12) 10482(9) 8822(6) 4471(7) 51(3)

C(13) 8307(10) 9471(6) 4868(7) 52(3)

C(14) 8185(11) 9163(6) 5818(7) 54(3)

C(15) 5356(9) 9061(6) 1812(7) 37(3)

C(16) 3982(8) 8724(6) 1585(7) 34(3)

C(17) 3966(9) 7393(6) 2553(6) 34(2)

C(18) 5112(8) 6821(6) 2664(6) 31(2)

C(19) 5056(9) 6025(5) 2095(6) 28(2)

C(20) 6173(9) 5799(6) 622(7) 41(3)

C(21) 7488(8) 6148(6) 874(7) 41(3)

N(1) 704(7) 6914(4) 8489(5) 26(2)

N(2) 1494(7) 8409(5) 7722(5) 29(2)

N(3) 3747(7) 7743(5) 7204(5) 35(2)

N(4) 2920(7) 6160(5) 8011(5) 35(2)

N(5) 9340(7) 7192(5) 3717(5) 34(2)

N(6) 9027(8) 8822(4) 4331(5) 34(2)

N(7) 7639(7) 8303(5) 5758(5) 36(2)

N(8) 7924(8) 6603(5) 5120(5) 43(2)

N(9) 4043(7) 7781(5) 1610(5) 28(2)

N(10) 5193(7) 6289(5) 1119(5) 33(2)

N(11) 7418(6) 7063(5) 719(5) 30(2)

N(12) 6243(7) 8632(5) 1205(5) 33(2)

O(2) 2884(5) 7612(4) 8964(4) 32(2)

O(4) 1394(6) 6914(4) 6641(4) 36(2)

O(6) 9998(5) 7671(4) 5391(4) 35(2)

O(8) 6852(6) 7880(4) 3951(4) 37(2)

O(10) 6233(5) 7275(4) 2350(3) 24(2)

O(12) 5137(5) 7580(4) -174(4) 43(2)

O(13) 7460(7) 8518(5) 9189(4) 64(2)

O(14) 8626(6) 8304(5) 7820(5) 58(2)

O(15) 7619(9) 9574(5) 8088(5) 97(3)

O(16) 6416(8) 8369(7) 7805(7) 110(4)

O(17) 3787(10) 563(6) 8676(6) 115(4)

O(18) 4938(10) 1174(7) 9857(8) 139(5)

O(19) 5009(9) -245(6) 9596(7) 128(4)

O(20) 3183(7) 343(5) 10175(5) 66(2)

O(21) 8928(6) 8416(5) 2189(5) 62(2)

O(22) 11098(8) 8274(6) 1818(6) 96(3)

O(23) 10192(10) 9570(6) 2100(7) 116(4)

O(24) 9555(9) 8815(7) 780(5) 93(4)

O(25) 5880(8) 6364(7) 7886(7) 124(4)

O(26) 7853(11) 6485(7) 7152(5) 129(4)

O(27) 7812(7) 6489(5) 8709(5) 64(2)

O(28) 7322(11) 5289(6) 7935(9) 139(4)

O(29) 770(10) 5234(7) 906(7) 118(4)

O(30) 233(8) 6430(6) 1661(6) 85(3)

O(31) 2292(6) 6317(4) 1017(4) 44(2)

O(32) 1750(7) 5567(6) 2319(5) 85(3)

O(33) 3001(13) 3594(11) 9835(13) 250(9)

O(34) 4838(12) 4286(6) 9648(7) 162(6)

O(35) 3917(12) 4183(7) 11045(7) 143(5)

O(36) 4776(16) 3052(10) 10328(9) 223(8)

Cl(1) 7524(3) 8712(2) 8231(2) 40(1)

Cl(2) 4258(2) 462(2) 9596(2) 42(1)

Cl(3) 9986(2) 8784(2) 1711(2) 37(1)

Cl(4) 7200(3) 6151(2) 7915(2) 49(1)

Cl(5) 1265(3) 5865(2) 1477(2) 43(1)

Cl(6) 4122(3) 3792(2) 10205(2) 55(1)

Co(1) 2226(1) 7277(1) 7812(1) 23(1)

Co(2) 8432(1) 7725(1) 4730(1) 24(1)

Co(3) 5724(1) 7451(1) 1115(1) 23(1)

________________________________________________________________________________

**Table C.** Bond lengths (Å) and angles (°) for [Co(L)(H_2_O)](ClO_4_)_2_ (**1**).

_____________________________________________________

C(1)-N(4) 1.475(11)

C(1)-C(2) 1.502(13)

C(2)-N(1) 1.488(11)

C(3)-N(1) 1.476(10)

C(3)-C(4) 1.534(11)

C(4)-O(2) 1.445(9)

C(4)-C(5) 1.524(12)

C(5)-N(2) 1.511(11)

C(6)-N(2) 1.481(11)

C(6)-C(7) 1.521(12)

C(7)-N(3) 1.474(12)

C(8)-N(8) 1.484(11)

C(8)-C(9) 1.505(13)

C(9)-N(5) 1.501(12)

C(10)-N(5) 1.494(11)

C(10)-C(11) 1.500(13)

C(11)-O(6) 1.453(10)

C(11)-C(12) 1.527(13)

C(12)-N(6) 1.513(11)

C(13)-C(14) 1.475(13)

C(13)-N(6) 1.510(12)

C(14)-N(7) 1.496(11)

C(15)-N(12) 1.460(11)

C(15)-C(16) 1.549(12)

C(16)-N(9) 1.518(11)

C(17)-C(18) 1.504(12)

C(17)-N(9) 1.512(10)

C(18)-O(10) 1.452(9)

C(18)-C(19) 1.525(12)

C(19)-N(10) 1.493(10)

C(20)-N(10) 1.485(11)

C(20)-C(21) 1.508(12)

C(21)-N(11) 1.490(11)

N(1)-Co(1) 1.963(7)

N(2)-Co(1) 1.975(8)

N(3)-Co(1) 1.970(7)

N(4)-Co(1) 1.952(7)

N(5)-Co(2) 1.962(8)

N(6)-Co(2) 1.959(8)

N(7)-Co(2) 1.956(7)

N(8)-Co(2) 1.966(8)

N(9)-Co(3) 1.968(8)

N(10)-Co(3) 1.947(8)

N(11)-Co(3) 1.957(6)

N(12)-Co(3) 1.976(7)

O(2)-Co(1) 1.874(6)

O(4)-Co(1) 1.979(6)

O(6)-Co(2) 1.866(5)

O(8)-Co(2) 1.983(6)

O(10)-Co(3) 1.883(5)

O(12)-Co(3) 1.969(6)

O(13)-Cl(1) 1.432(6)

O(14)-Cl(1) 1.454(7)

O(15)-Cl(1) 1.405(8)

O(16)-Cl(1) 1.403(8)

O(17)-Cl(2) 1.424(9)

O(18)-Cl(2) 1.392(9)

O(19)-Cl(2) 1.377(9)

O(20)-Cl(2) 1.422(7)

O(21)-Cl(3) 1.437(7)

O(22)-Cl(3) 1.418(8)

O(23)-Cl(3) 1.399(9)

O(24)-Cl(3) 1.418(7)

O(25)-Cl(4) 1.407(8)

O(26)-Cl(4) 1.417(9)

O(27)-Cl(4) 1.413(7)

O(28)-Cl(4) 1.391(10)

O(29)-Cl(5) 1.401(9)

O(30)-Cl(5) 1.431(8)

O(31)-Cl(5) 1.462(6)

O(32)-Cl(5) 1.397(7)

O(33)-Cl(6) 1.307(11)

O(34)-Cl(6) 1.364(9)

O(35)-Cl(6) 1.394(10)

O(36)-Cl(6) 1.378(13)

N(4)-C(1)-C(2) 107.2(8)

N(1)-C(2)-C(1) 108.1(7)

N(1)-C(3)-C(4) 107.9(7)

O(2)-C(4)-C(5) 106.3(7)

O(2)-C(4)-C(3) 106.3(7)

C(5)-C(4)-C(3) 113.2(7)

N(2)-C(5)-C(4) 106.2(7)

N(2)-C(6)-C(7) 106.9(7)

N(3)-C(7)-C(6) 107.7(8)

N(8)-C(8)-C(9) 108.2(8)

N(5)-C(9)-C(8) 108.6(8)

N(5)-C(10)-C(11) 109.7(8)

O(6)-C(11)-C(10) 106.2(8)

O(6)-C(11)-C(12) 103.7(8)

C(10)-C(11)-C(12) 113.8(8)

N(6)-C(12)-C(11) 108.0(8)

C(14)-C(13)-N(6) 107.9(8)

C(13)-C(14)-N(7) 107.2(8)

N(12)-C(15)-C(16) 106.9(8)

N(9)-C(16)-C(15) 107.8(7)

C(18)-C(17)-N(9) 107.0(7)

O(10)-C(18)-C(17) 106.9(7)

O(10)-C(18)-C(19) 105.7(7)

C(17)-C(18)-C(19) 115.8(7)

N(10)-C(19)-C(18) 105.9(7)

N(10)-C(20)-C(21) 107.7(7)

N(11)-C(21)-C(20) 106.9(7)

C(3)-N(1)-C(2) 114.5(7)

C(3)-N(1)-Co(1) 107.5(5)

C(2)-N(1)-Co(1) 107.8(6)

C(6)-N(2)-C(5) 114.1(7)

C(6)-N(2)-Co(1) 109.0(6)

C(5)-N(2)-Co(1) 107.2(5)

C(7)-N(3)-Co(1) 109.1(6)

C(1)-N(4)-Co(1) 107.7(6)

C(10)-N(5)-C(9) 114.9(8)

C(10)-N(5)-Co(2) 105.5(6)

C(9)-N(5)-Co(2) 108.4(6)

C(13)-N(6)-C(12) 115.5(8)

C(13)-N(6)-Co(2) 107.8(7)

C(12)-N(6)-Co(2) 106.1(5)

C(14)-N(7)-Co(2) 108.6(6)

C(8)-N(8)-Co(2) 108.4(6)

C(17)-N(9)-C(16) 115.5(7)

C(17)-N(9)-Co(3) 106.6(5)

C(16)-N(9)-Co(3) 107.3(5)

C(20)-N(10)-C(19) 113.1(7)

C(20)-N(10)-Co(3) 108.2(6)

C(19)-N(10)-Co(3) 108.0(5)

C(21)-N(11)-Co(3) 108.1(5)

C(15)-N(12)-Co(3) 108.7(5)

C(4)-O(2)-Co(1) 101.1(4)

C(11)-O(6)-Co(2) 101.4(5)

C(18)-O(10)-Co(3) 99.7(4)

O(16)-Cl(1)-O(15) 112.4(6)

O(16)-Cl(1)-O(13) 106.8(6)

O(15)-Cl(1)-O(13) 111.3(5)

O(16)-Cl(1)-O(14) 106.3(5)

O(15)-Cl(1)-O(14) 109.1(5)

O(13)-Cl(1)-O(14) 110.9(4)

O(19)-Cl(2)-O(18) 113.5(7)

O(19)-Cl(2)-O(20) 109.7(5)

O(18)-Cl(2)-O(20) 110.2(6)

O(19)-Cl(2)-O(17) 106.0(7)

O(18)-Cl(2)-O(17) 108.8(7)

O(20)-Cl(2)-O(17) 108.5(5)

O(23)-Cl(3)-O(22) 111.3(6)

O(23)-Cl(3)-O(24) 113.3(6)

O(22)-Cl(3)-O(24) 111.2(6)

O(23)-Cl(3)-O(21) 106.7(5)

O(22)-Cl(3)-O(21) 109.4(5)

O(24)-Cl(3)-O(21) 104.5(5)

O(28)-Cl(4)-O(25) 109.3(7)

O(28)-Cl(4)-O(27) 109.1(6)

O(25)-Cl(4)-O(27) 110.3(6)

O(28)-Cl(4)-O(26) 110.5(8)

O(25)-Cl(4)-O(26) 111.3(7)

O(27)-Cl(4)-O(26) 106.3(6)

O(32)-Cl(5)-O(29) 113.0(6)

O(32)-Cl(5)-O(30) 107.9(5)

O(29)-Cl(5)-O(30) 107.9(6)

O(32)-Cl(5)-O(31) 108.8(4)

O(29)-Cl(5)-O(31) 110.4(5)

O(30)-Cl(5)-O(31) 108.8(5)

O(33)-Cl(6)-O(34) 112.6(10)

O(33)-Cl(6)-O(36) 105.7(12)

O(34)-Cl(6)-O(36) 108.0(8)

O(33)-Cl(6)-O(35) 108.7(10)

O(34)-Cl(6)-O(35) 110.8(6)

O(36)-Cl(6)-O(35) 111.0(8)

O(2)-Co(1)-N(4) 90.4(3)

O(2)-Co(1)-N(1) 85.2(3)

N(4)-Co(1)-N(1) 87.1(3)

O(2)-Co(1)-N(3) 90.8(3)

N(4)-Co(1)-N(3) 97.1(3)

N(1)-Co(1)-N(3) 174.3(3)

O(2)-Co(1)-N(2) 85.7(3)

N(4)-Co(1)-N(2) 175.2(3)

N(1)-Co(1)-N(2) 89.8(3)

N(3)-Co(1)-N(2) 85.8(3)

O(2)-Co(1)-O(4) 175.4(3)

N(4)-Co(1)-O(4) 90.5(3)

N(1)-Co(1)-O(4) 90.4(3)

N(3)-Co(1)-O(4) 93.5(3)

N(2)-Co(1)-O(4) 93.2(3)

O(6)-Co(2)-N(7) 90.1(3)

O(6)-Co(2)-N(6) 85.4(3)

N(7)-Co(2)-N(6) 86.5(3)

O(6)-Co(2)-N(5) 86.7(3)

N(7)-Co(2)-N(5) 175.9(3)

N(6)-Co(2)-N(5) 90.7(3)

O(6)-Co(2)-N(8) 92.4(3)

N(7)-Co(2)-N(8) 95.5(3)

N(6)-Co(2)-N(8) 177.1(4)

N(5)-Co(2)-N(8) 87.2(3)

O(6)-Co(2)-O(8) 173.9(3)

N(7)-Co(2)-O(8) 91.4(3)

N(6)-Co(2)-O(8) 88.7(3)

N(5)-Co(2)-O(8) 91.5(3)

N(8)-Co(2)-O(8) 93.4(3)

O(10)-Co(3)-N(10) 85.7(3)

O(10)-Co(3)-N(11) 90.0(3)

N(10)-Co(3)-N(11) 87.1(3)

O(10)-Co(3)-N(9) 85.5(3)

N(10)-Co(3)-N(9) 90.4(3)

N(11)-Co(3)-N(9) 174.9(3)

O(10)-Co(3)-O(12) 176.9(3)

N(10)-Co(3)-O(12) 91.3(3)

N(11)-Co(3)-O(12) 90.7(3)

N(9)-Co(3)-O(12) 93.7(3)

O(10)-Co(3)-N(12) 90.6(3)

N(10)-Co(3)-N(12) 175.9(3)

N(11)-Co(3)-N(12) 94.8(3)

N(9)-Co(3)-N(12) 87.5(3)

O(12)-Co(3)-N(12) 92.3(3)

_____________________________________________________________

**Table D.** Anisotropic displacement parameters (Å2 × 103) for [Co(L)(H_2_O)](ClO_4_)_2_ (**1**). The anisotropic displacement factor exponent takes the form: -2π2[ h2a*2U11 + ... + 2 h k a* b* U12 ]

______________________________________________________________________________

U11 U22 U33 U23 U13 U12

______________________________________________________________________________

C(1) 60(8) 29(7) 39(6) 3(5) 2(6) -4(5)

C(2) 48(7) 28(7) 40(6) 8(5) -2(5) -9(5)

C(3) 24(6) 45(7) 19(5) -3(5) 3(4) 2(5)

C(4) 29(6) 32(6) 19(5) -11(4) -8(4) 16(5)

C(5) 34(7) 31(7) 49(7) -8(5) -1(5) -9(5)

C(6) 40(7) 35(7) 41(7) 11(5) -1(5) 9(5)

C(7) 36(7) 43(8) 53(7) 7(5) -1(5) -8(5)

C(8) 97(11) 35(7) 39(8) 11(6) 14(7) 2(7)

C(9) 81(9) 33(8) 47(7) -9(6) 8(6) 4(6)

C(10) 43(7) 43(7) 38(6) 6(5) 9(5) 14(5)

C(11) 25(6) 72(9) 36(6) 8(6) -14(5) 1(5)

C(12) 43(7) 63(9) 46(7) 21(6) -29(5) -24(6)

C(13) 65(8) 32(7) 57(8) -8(6) -9(6) 5(6)

C(14) 94(9) 30(7) 38(7) -9(5) 14(6) 1(6)

C(15) 37(7) 26(6) 47(7) 4(5) 1(6) 5(5)

C(16) 26(7) 31(7) 44(6) 2(5) 2(5) -3(5)

C(17) 40(6) 38(6) 24(6) -4(5) 5(5) -12(5)

C(18) 27(6) 46(7) 21(5) 11(5) -4(5) -15(5)

C(19) 30(6) 13(6) 43(6) 9(4) 7(5) 3(4)

C(20) 45(7) 34(7) 45(7) -18(5) 8(6) -16(5)

C(21) 24(6) 53(8) 47(7) -17(6) 13(5) 8(5)

N(1) 25(5) 29(5) 24(5) -6(4) -5(4) -2(4)

N(2) 21(5) 37(5) 28(5) 6(4) -4(4) -14(4)

N(3) 21(5) 54(6) 30(5) 0(4) -4(4) -5(4)

N(4) 30(5) 36(5) 39(5) -9(4) -5(4) 0(4)

N(5) 23(5) 51(6) 28(5) 13(4) 1(4) -1(4)

N(6) 44(5) 39(5) 18(4) 7(4) -20(4) 5(5)

N(7) 24(5) 59(6) 24(5) -6(4) -5(4) 2(4)

N(8) 48(6) 45(6) 38(5) 6(4) 5(4) -12(5)

N(9) 30(5) 27(5) 27(5) -1(4) 3(4) -10(4)

N(10) 26(5) 41(6) 31(5) -3(4) -5(4) -4(4)

N(11) 17(5) 53(6) 20(4) -6(4) -1(3) -2(4)

N(12) 22(5) 38(5) 39(5) 3(4) 3(4) -14(4)

O(2) 20(4) 50(5) 26(4) -7(3) -4(3) 8(3)

O(4) 24(4) 57(5) 25(4) 5(3) -11(3) 5(3)

O(6) 28(4) 49(5) 26(4) 9(3) 0(3) 7(3)

O(8) 29(4) 58(5) 22(4) -7(3) -7(3) 6(3)

O(10) 21(4) 29(4) 22(4) -2(3) 3(3) -3(3)

O(12) 17(4) 80(6) 31(4) 7(4) -7(3) -13(4)

O(13) 85(6) 77(6) 32(5) 22(4) 24(4) 12(5)

O(14) 23(5) 97(7) 54(5) 18(4) 3(4) 14(4)

O(15) 173(11) 47(6) 72(7) 18(5) 38(6) 16(6)

O(16) 26(5) 167(11) 136(9) -73(8) -11(5) 1(6)

O(17) 146(9) 145(10) 54(6) 38(6) -18(6) -23(8)

O(18) 131(9) 130(10) 158(10) -55(8) 38(8) -99(8)

O(19) 101(8) 125(9) 161(10) 56(8) 45(7) 68(7)

O(20) 36(5) 95(7) 69(5) 6(5) 23(4) 0(5)

O(21) 28(4) 95(7) 63(5) 30(4) 5(4) -10(4)

O(22) 47(5) 113(8) 129(8) 49(6) 3(5) 24(5)

O(23) 120(8) 81(8) 148(10) -52(7) 19(7) -53(6)

O(24) 94(7) 152(11) 34(5) 15(5) -1(5) 42(7)

O(25) 36(6) 187(12) 148(10) -3(8) -29(6) 31(7)

O(26) 179(11) 189(12) 20(5) 13(6) 37(6) -1(9)

O(27) 57(5) 91(7) 44(5) -23(4) -1(4) -17(4)

O(28) 134(10) 57(8) 223(12) -24(8) -62(9) -12(7)

O(29) 127(9) 126(9) 103(8) -46(7) 25(7) -81(7)

O(30) 59(6) 94(8) 103(7) 35(6) 6(5) 23(5)

O(31) 34(4) 59(5) 38(4) 16(4) 3(3) -6(4)

O(32) 61(5) 130(8) 64(6) 62(5) -12(4) -9(5)

O(33) 114(12) 300(20) 330(20) -11(17) -83(12) -126(13)

O(34) 274(15) 128(10) 88(8) -60(7) 103(9) -125(10)

O(35) 237(13) 111(9) 84(7) -3(7) 97(8) 5(9)

O(36) 300(20) 218(16) 152(12) 57(11) 103(12) 159(15)

Cl(1) 36(2) 50(2) 34(2) -4(1) -2(1) -1(1)

Cl(2) 37(2) 45(2) 44(2) 7(1) 0(1) -1(1)

Cl(3) 35(2) 44(2) 33(2) 6(1) -1(1) -2(1)

Cl(4) 47(2) 49(2) 49(2) -5(2) -10(2) -7(2)

Cl(5) 35(2) 58(2) 35(2) 6(2) -5(1) -14(2)

Cl(6) 47(2) 69(2) 50(2) 1(2) 8(2) -16(2)

Co(1) 16(1) 31(1) 24(1) 1(1) 0(1) 2(1)

Co(2) 24(1) 31(1) 18(1) -1(1) -4(1) 2(1)

Co(3) 17(1) 32(1) 20(1) 0(1) 0(1) -2(1)

**Table E.** Selected intermolecular interactions parameters for [Co(L)(H_2_O)](ClO_4_)_2_ (**1**) (A, °).

A H B d(A-B) d(A-H) d(H...B) (A-H...B) B...H...B* A'..H..A"

N(1) -H(1) ..O(14)ii 0.91 2.52 3.234(10) 136

N(1) -H(1) ..O(27)ii 0.91 2.55 3.089(10) 118' 82' 336.00

O(8) -H(1W) ..O(10)vi 0.93 1.66 2.591(8) 175

N(2) -H(2) ..O(14)ii 0.91 2.17 2.975(10) 147

O(8) -H(2W) ..O(18)vi 0.84 2.20 2.842(13) 133

N(3) -H(3) ..O(16) 0.90 2.41 3.046(11) 128

N(3) -H(3) ..O(25) 0.90 2.48 3.265(13) 146' 84' 358.00

O(4) -H(3W) ..O(33) 0.90 2.41 3.244(18) 153

N(4) -H(4A) ..O(32) 0.90 2.55 3.183(12) 128

N(4) -H(4A) ..O(35) 0.90 2.42 3.111(13) 134' 96' 358.00

N(4) -H(4B) ..O(25) 0.90 2.25 3.086(11) 155

N(3) -H(4N) ..O(36) 0.90 2.38 3.216(16) 154

O(4) -H(4W) ..O(6)i 0.99 1.79 2.599(8) 136

O(12)-H(5W) ..O(13) 1.10 2.05 2.999(9) 143

O(12)-H(5W) ..O(16) 1.10 2.46 3.490(12) 155' 60' 358.00

O(12)-H(6W) ..O(2) 0.89 1.78 2.620(8) 155

N(6) -H(7) ..O(21)vi 0.91 2.36 3.182(10) 151

N(5) -H(8) ..O(21)vi 0.91 2.13 2.992(11) 158

N(7) -H(9) ..O(18)vi 0.90 2.36 3.170(13) 151

N(7) -H(10) ..O(14)iii 0.90 2.41 3.147(9) 139

N(8) -H(11N)..O(26)iii 0.90 2.08 2.965(10) 167

N(8) -H(12) ..O(36)iii 0.90 2.55 3.320(18) 144

N(9) -H(13) ..O(31)iii 0.91 2.34 3.081(10) 139

N(10)-H(14) ..O(31)iii 0.91 2.22 2.999(10) 143

N(11)-H(15) ..O(13) 0.90 2.55 3.232(10) 133

N(11)-H(15) ..O(27) 0.90 2.36 3.104(9) 140' 86' 359.00

N(11)-H(16) ..O(21) 0.90 2.58 3.405(10) 154

N(12)-H(17) ..O(21) 0.90 2.24 3.114(10) 163

N(12)-H(18N)..O(13) 0.90 2.56 3.223(10) 131

N(12)-H(18N)..O(19)v 0.90 2.41 3.199(12) 146' 82' 359.00

C(1) -H(1A) ..O(31)iii 0.97 2.49 3.387(12) 154

C(1) -H(1B) ..O(32) 0.97 2.48 3.048(13) 117

C(3) -H(3A) ..O(31)iii 0.97 2.51 3.383(10) 150

C(6) -H(6A) ..O(23)iv 0.97 2.52 3.259(14) 133

A H B d(A-B) d(A-H) d(H...B) (A-H...B) B...H...B* A'..H..A"

C(10)-H(10A)..O(22)vi 0.97 2.53 3.428(13) 153

C(10)-H(10B)..O(33)vii 0.97 2.28 3.055(18) 137

C(13)-H(13A)..O(24)viii 0.97 2.46 3.308(14) 146

C(13)-H(13B)..O(15)viii 0.97 2.60 3.080(13) 111

C(16)-H(16A)..O(17) 0.97 2.56 3.262(13) 130

C(16)-H(16A)..O(22)ii 0.97 2.59 3.093(12) 113' 112' 355.00

C(18)-H(18) ..O(34)ix 0.98 2.53 3.409(14) 150

C(19)-H(19A)..O(28)iii 0.97 2.40 3.364(14) 172

C(19)-H(19B)..O(32)iii 0.97 2.60 3.517(12) 158

C(20)-H(20B)..O(34)iii 0.97 2.59 3.121(15) 114

*Code: i = -1+x,1-y,-1/2+z; ii = -1+x,y,z; iii = x,1-y,1/2+z; iv = -1+x,-y,-1/2+z; v = x,-y,-1/2+z; vi = x,1-y,-1/2+z; vii= 1+x,1-y,1/2+z; viii= x,1+y,z; ix= x,y,1+z*
