# Supplementary material for: Synthesis and Characterization of Cobalt(III), Nickel(II) and Copper(II) Mononuclear Complexes with the Ligand 1,3-bis[(2-aminoethyl)amino]-2-propanol and Their Catalase-Like Activity
Source: PLoS One. 2015 Sep 17;10(9):e0137926. doi: 10.1371/journal.pone.0137926 (PMC4574563; doi:10.1371/journal.pone.0137926)
Supplement: S3 File — (DOCX) [file pone.0137926.s006.docx]

Supporting Information for

#### Synthesis and Characterization of Cobalt(III), Nickel(II) and Copper(II) Mononuclear Complexes with the Ligand 1,3-bis[(2-aminoethyl)amino]-2-propanol and their Catalase-like Activity.

Bianca M. Pires^1¶^, Daniel M. Silva^1^, Lorenzo C. Visentin^2&^, Bernardo L. Rodrigues^3&^, Nakédia M. F. Carvalho^1¶,#a^, Roberto B. Faria^1¶*^

^1^ Instituto de Química, Universidade Federal do Rio de Janeiro, Rio de Janeiro, Rio de Janeiro, Brazil

^2^ NanoBusiness Informação e Inovação Ltda., Rio de Janeiro, Rio de Janeiro, Brazil

^3^ Departamento de Química, Universidade Federal de Minas Gerais, Belo Horizonte, Minas Gerais, Brazil

^#a^ Current Address: Instituto de Química, Universidade do Estado do Rio de Janeiro, Rio de Janeiro, Rio de Janeiro, Brazil

* Corresponding author

E-mail: [faria@iq.ufrj.br](mailto:faria@iq.ufrj.br) (RBF)

**Table A.** Crystal data and structure refinement for [Ni(HL)](ClO_4_)_2_ (**2**).

Identification code [Ni(L)](ClO_4_)_2_

Empirical formula C7 H20 Cl2 N4 Ni O9

Formula weight 433.86

Temperature 293(2) K

Wavelength 0.71073 Å

Crystal system monoclinic

Space group *P*2_1_/*n*

Unit cell dimensions *a* = 9.488(5) Å *α* = 90°.

*b* = 13.994(5) Å *β* = 93.450(5)°.

*c* = 11.828(5) Å *γ* = 90°.

Volume 1567.6(12) Å3

*Z* 4

Density (calculated) 1.838 Mg/m3

Absorption coefficient 1.630 mm-1

*F*(000) 896

Crystal size 0.062 x 0.200 x 0.212 mm3

Theta range for data collection 3.38 to 27.50°.

Index ranges -12<=*h*<=12, -18<=*k*<=18, -15<=*l*<=14

Reflections collected 19990

Independent reflections 3208 [*R*(int) = 0.1143]

Completeness to theta = 26.50° 87.8 %

Absorption correction None

Refinement method Full-matrix least-squares on *F*2

Data / restraints / parameters 3208 / 0 / 208

Goodness-of-fit on *F*2 1.035

Final *R* indices [*I*>2sigma(I)] *R*1 = 0.0541, *wR*2 = 0.0930

R indices (all data) *R*1 = 0.1163, *wR*2 = 0.1104

Largest diff. peak and hole 0.406 and -0.4029e.Å-3

**Table B.** Atomic coordinates (× 104) and equivalent isotropic displacement parameters (Å2 × 103) for [Ni(HL)](ClO_4_)_2_ (**2**). U(eq) is defined as one third of the trace of the orthogonalized Uij tensor.

________________________________________________________________________________

x y z U(eq)

________________________________________________________________________________

Ni 2554(1) 1124(1) 2642(1) 26(1)

Cl(1) 3028(2) 1217(1) -1494(1) 39(1)

Cl(2) 2503(1) -1410(1) 3423(1) 35(1)

O(1) 1930(6) 2988(3) 4831(3) 75(2)

O(11) 4134(6) 971(4) -665(4) 85(2)

O(12) 1709(5) 1198(3) -994(4) 65(1)

O(13) 3283(5) 2156(2) -1899(4) 66(1)

O(14) 3028(5) 573(3) -2418(3) 61(1)

O(21) 3731(6) -1391(4) 2835(5) 93(2)

O(22) 1328(6) -1370(3) 2611(4) 72(1)

O(23) 2448(6) -2276(2) 4031(4) 72(1)

O(24) 2471(6) -617(3) 4148(4) 78(2)

N(1) 1404(6) 560(3) 1443(4) 50(1)

N(2) 832(5) 1481(3) 3295(4) 39(1)

N(3) 3728(5) 1735(3) 3801(3) 36(1)

N(4) 4242(5) 804(3) 1936(4) 44(1)

C(1) -55(8) 895(4) 1510(7) 76(2)

C(2) -304(7) 908(4) 2726(7) 69(2)

C(3) 773(8) 1458(4) 4552(6) 64(2)

C(4) 1920(9) 2008(4) 5161(5) 64(2)

C(5) 3359(7) 1617(4) 4994(4) 52(2)

C(6) 5218(6) 1453(4) 3652(5) 51(1)

C(7) 5399(6) 1427(4) 2414(6) 53(2)

________________________________________________________________________________

**Table C.** Bond lengths (Å) and angles (°) for [Ni(HL)](ClO_4_)_2_ (**2**).

_____________________________________________________

Ni-N(4) 1.903(4)

Ni-N(1) 1.907(5)

Ni-N(2) 1.915(4)

Ni-N(3) 1.915(4)

Cl(1)-O(12) 1.416(4)

Cl(1)-O(14) 1.417(3)

Cl(1)-O(13) 1.424(4)

Cl(1)-O(11) 1.435(5)

Cl(2)-O(21) 1.392(4)

Cl(2)-O(24) 1.404(4)

Cl(2)-O(23) 1.413(4)

Cl(2)-O(22) 1.429(5)

O(1)-C(4) 1.427(6)

O(1)-HO1 0.8200

N(1)-C(1) 1.468(8)

N(1)-HN1A 0.9000

N(1)-HN1B 0.9000

N(2)-C(2) 1.473(8)

N(2)-C(3) 1.492(7)

N(2)-HN2 0.9100

N(3)-C(5) 1.483(6)

N(3)-C(6) 1.489(7)

N(3)-HN3 0.9100

N(4)-C(7) 1.487(8)

N(4)-HN4A 0.9000

N(4)-HN4B 0.9000

C(1)-C(2) 1.472(10)

C(1)-H(1A) 0.9700

C(1)-H(1B) 0.9700

C(2)-H(2A) 0.9700

C(2)-H(2B) 0.9700

C(3)-C(4) 1.484(10)

C(3)-H(3A) 0.9700

C(3)-H(3B) 0.9700

C(4)-C(5) 1.495(9)

C(4)-H(4) 0.9800

C(5)-H(5A) 0.9700

C(5)-H(5B) 0.9700

C(6)-C(7) 1.485(8)

C(6)-H(6A) 0.9700

C(6)-H(6B) 0.9700

C(7)-H(7A) 0.9700

C(7)-H(7B) 0.9700

N(4)-Ni-N(1) 92.0(2)

N(4)-Ni-N(2) 177.40(19)

N(1)-Ni-N(2) 86.8(2)

N(4)-Ni-N(3) 87.18(19)

N(1)-Ni-N(3) 177.44(16)

N(2)-Ni-N(3) 93.92(19)

O(12)-Cl(1)-O(14) 110.6(3)

O(12)-Cl(1)-O(13) 109.3(3)

O(14)-Cl(1)-O(13) 108.7(2)

O(12)-Cl(1)-O(11) 109.7(3)

O(14)-Cl(1)-O(11) 109.9(3)

O(13)-Cl(1)-O(11) 108.6(3)

O(21)-Cl(2)-O(24) 109.9(3)

O(21)-Cl(2)-O(23) 109.1(3)

O(24)-Cl(2)-O(23) 111.4(3)

O(21)-Cl(2)-O(22) 107.9(3)

O(24)-Cl(2)-O(22) 109.6(3)

O(23)-Cl(2)-O(22) 108.9(3)

C(4)-O(1)-HO1 109.5

C(1)-N(1)-Ni 109.1(4)

C(1)-N(1)-HN1A 109.9

Ni-N(1)-HN1A 109.9

C(1)-N(1)-HN1B 109.9

Ni-N(1)-HN1B 109.9

HN1A-N(1)-HN1B 108.3

C(2)-N(2)-C(3) 111.9(5)

C(2)-N(2)-Ni 107.0(3)

C(3)-N(2)-Ni 118.7(4)

C(2)-N(2)-HN2 106.1

C(3)-N(2)-HN2 106.1

Ni-N(2)-HN2 106.1

C(5)-N(3)-C(6) 111.3(5)

C(5)-N(3)-Ni 118.1(4)

C(6)-N(3)-Ni 108.2(3)

C(5)-N(3)-HN3 106.1

C(6)-N(3)-HN3 106.1

Ni-N(3)-HN3 106.1

C(7)-N(4)-Ni 108.5(3)

C(7)-N(4)-HN4A 110.0

Ni-N(4)-HN4A 110.0

C(7)-N(4)-HN4B 110.0

Ni-N(4)-HN4B 110.0

HN4A-N(4)-HN4B 108.4

N(1)-C(1)-C(2) 105.3(6)

N(1)-C(1)-H(1A) 110.7

C(2)-C(1)-H(1A) 110.7

N(1)-C(1)-H(1B) 110.7

C(2)-C(1)-H(1B) 110.7

H(1A)-C(1)-H(1B) 108.8

C(1)-C(2)-N(2) 107.3(5)

C(1)-C(2)-H(2A) 110.3

N(2)-C(2)-H(2A) 110.3

C(1)-C(2)-H(2B) 110.3

N(2)-C(2)-H(2B) 110.3

H(2A)-C(2)-H(2B) 108.5

C(4)-C(3)-N(2) 113.8(5)

C(4)-C(3)-H(3A) 108.8

N(2)-C(3)-H(3A) 108.8

C(4)-C(3)-H(3B) 108.8

N(2)-C(3)-H(3B) 108.8

H(3A)-C(3)-H(3B) 107.7

O(1)-C(4)-C(3) 112.5(6)

O(1)-C(4)-C(5) 107.1(6)

C(3)-C(4)-C(5) 113.3(5)

O(1)-C(4)-H(4) 107.9

C(3)-C(4)-H(4) 107.9

C(5)-C(4)-H(4) 107.9

N(3)-C(5)-C(4) 110.8(5)

N(3)-C(5)-H(5A) 109.5

C(4)-C(5)-H(5A) 109.5

N(3)-C(5)-H(5B) 109.5

C(4)-C(5)-H(5B) 109.5

H(5A)-C(5)-H(5B) 108.1

C(7)-C(6)-N(3) 106.9(5)

C(7)-C(6)-H(6A) 110.3

N(3)-C(6)-H(6A) 110.3

C(7)-C(6)-H(6B) 110.3

N(3)-C(6)-H(6B) 110.3

H(6A)-C(6)-H(6B) 108.6

C(6)-C(7)-N(4) 105.2(4)

C(6)-C(7)-H(7A) 110.7

N(4)-C(7)-H(7A) 110.7

C(6)-C(7)-H(7B) 110.7

N(4)-C(7)-H(7B) 110.7

H(7A)-C(7)-H(7B) 108.8

_____________________________________________________________

Symmetry transformations used to generate equivalent atoms:

**Table D.** Anisotropic displacement parameters (Å2 × 103) for [Ni(HL)](ClO_4_)_2_ (**2**). The anisotropic

displacement factor exponent takes the form: -2π2[ h2a*2U11 + ... + 2 h k a* b* U12 ]

_____________________________________________________________________________

U11 U22 U33 U23 U13 U12

______________________________________________________________________________

Ni 26(1) 24(1) 27(1) 1(1) -1(1) 0(1)

Cl(1) 45(1) 36(1) 36(1) -2(1) 4(1) -2(1)

Cl(2) 40(1) 26(1) 39(1) 0(1) -2(1) 2(1)

O(1) 123(5) 47(2) 56(2) -7(2) 2(3) 31(2)

O(11) 92(4) 114(4) 46(2) 2(3) -12(3) 34(3)

O(12) 60(3) 67(3) 71(3) -7(2) 25(3) -9(2)

O(13) 77(4) 40(2) 81(3) 6(2) 12(3) -9(2)

O(14) 79(3) 52(2) 51(2) -19(2) 5(2) -7(2)

O(21) 58(4) 107(4) 118(5) -7(3) 42(4) -5(3)

O(22) 76(4) 43(2) 92(3) -1(2) -40(3) 0(2)

O(23) 116(4) 32(2) 66(2) 13(2) -17(3) 1(2)

O(24) 108(4) 45(2) 77(3) -26(2) -16(3) 9(3)

N(1) 73(4) 36(2) 38(2) 4(2) -15(2) -14(2)

N(2) 32(3) 28(2) 58(3) 7(2) 6(2) 3(2)

N(3) 41(3) 27(2) 37(2) -1(2) -6(2) 0(2)

N(4) 45(3) 45(2) 43(2) -4(2) 10(2) 7(2)

C(1) 67(5) 39(3) 113(6) 3(3) -52(4) -9(3)

C(2) 31(3) 37(3) 138(7) -3(3) 1(4) -6(2)

C(3) 71(5) 56(3) 72(4) 16(3) 45(4) 27(3)

C(4) 111(6) 53(3) 30(3) 1(2) 11(4) 29(4)

C(5) 82(5) 44(3) 27(3) -5(2) -11(3) 14(3)

C(6) 34(3) 45(3) 72(4) 6(3) -15(3) -2(2)

C(7) 34(3) 46(3) 82(4) 14(3) 11(3) 4(3)

______________________________________________________________________________

**Table E.** Selected intermolecular interactions parameters for [Ni(HL)](ClO_4_)_2_ (**2**) (A, °).

A H B d(A—B) d(A-H) d(H...B) ∠(A–H...B)

O1 HO1 O11i 3.052(8) 0.82 2.44 131.7

N4 HN4b O11 3.081(6) 0.90 2.19 170.8

N1 HN1a O12 3.048(6) 0.90 2.19 159.4

C1 H1a O12ii 3.361(7) 0.97 2.51 146.0

C2 H2b O13i 3.067(7) 0.97 2.39 126.8

N2 Hn2 O13i 3.078(6) 0.91 2.49 122.8

N4 HN4a O14iii 3.251(7) 0.90 2.62 127.9

N2 Hn2 O21iv 3.300(6) 0.91 2.46 153.3

O1 HO1 O21iv 3.295(7) 0.82 2.67 133.9

N4 HN4a O21 3.297(6) 0.90 2.50 147.6

N1 HN1b O22 3.036(5) 0.90 2.24 147.9

N3 HN3 O22iv 3.133(5) 0.91 2.31 150.4

C3 H3b O24 3.368(7) 0.97 2.67 129.5

C5 H5b O24 3.374(7) 0.97 2.63 133.5

Symmetry operations: (i) x-1/2, -y+1/2, z+1/2; (ii) -x, -y, -z; (iii) -x+1, -y, -z;

(iv) -x+1/2, y+1/2, -z+1/2
