# Supplementary material for: Synthesis and Characterization of Cobalt(III), Nickel(II) and Copper(II) Mononuclear Complexes with the Ligand 1,3-bis[(2-aminoethyl)amino]-2-propanol and Their Catalase-Like Activity
Source: PLoS One. 2015 Sep 17;10(9):e0137926. doi: 10.1371/journal.pone.0137926 (PMC4574563; doi:10.1371/journal.pone.0137926)
Supplement: S1 Table — (DOCX) [file pone.0137926.s007.docx]

Supporting Information for

#### Synthesis and Characterization of Cobalt(III), Nickel(II) and Copper(II) Mononuclear Complexes with the Ligand 1,3-bis[(2-aminoethyl)amino]-2-propanol and their Catalase-like Activity.

Bianca M. Pires^1¶^, Daniel M. Silva^1^, Lorenzo C. Visentin^2&^, Bernardo L. Rodrigues^3&^, Nakédia M. F. Carvalho^1¶,#a^, Roberto B. Faria^1¶*^

^1^ Instituto de Química, Universidade Federal do Rio de Janeiro, Rio de Janeiro, Rio de Janeiro, Brazil

^2^ NanoBusiness Informação e Inovação Ltda., Rio de Janeiro, Rio de Janeiro, Brazil

^3^ Departamento de Química, Universidade Federal de Minas Gerais, Belo Horizonte, Minas Gerais, Brazil

^#a^ Current Address: Instituto de Química, Universidade do Estado do Rio de Janeiro, Rio de Janeiro, Rio de Janeiro, Brazil

* Corresponding author

E-mail: [faria@iq.ufrj.br](mailto:faria@iq.ufrj.br) (RBF)

**S1 Table.** Yield of cyclohexanol and cyclohexanone after 24 hours in CH_3_CN or H_2_O as solvent.

| **Cyclohexane oxidation (%)** | | |
| --- | --- | --- |
| **Complex** | **CH_3_CN** | **H_2_O** |
| (**1**) | 0.37 | 0 |
| (**2**) | 0.15 | 0 |
| (**3**) | 1.79 | 0.29 |
| [Fe_4_(μ-O)(μ-OH)(μOAc)_4_(L)_2_](ClO_4_)_3_ | 1.5 | - |
